# Transcriptome and metabolomic analysis to reveal the browning spot formation of ‘Huangguan’ pear

**DOI:** 10.1186/s12870-021-03049-8

**Published:** 2021-07-03

**Authors:** Qi Wang, Xinyi Wu, Li Liu, Daozhi Yao, Jinchao Li, Jie Fang, Xiaonan Chen, Liwu Zhu, Pu Liu, Zhenfeng Ye, Bing Jia, Wei Heng

**Affiliations:** College of Horticulture, Anhui Agricultural University, Hefei, Anhui 230036 P. R. China

**Keywords:** ‘Huangguan’ pear, Browning disorder, Transcriptome, Metabolomic, Molecular mechanism

## Abstract

**Background:**

Browning spot (BS) disorders seriously affect the appearance quality of ‘Huangguan’ pear and cause economic losses. Many studies on BS have mainly focused on physiological and biochemical aspects, and the molecular mechanism remains unclear.

**Results:**

In the present study, the structural characteristics of ‘Huangguan’ pear with BS were observed via scanning electron microscopy (SEM), the water loss and brown spots were evaluated, and transcriptomic and metabolomics analyses were conducted to reveal the molecular mechanism underlying ‘Huangguan’ pear skin browning disorder. The results showed that the occurrence of BS was accompanied by a decrease in the wax layer and an increase in lignified cells. Genes related to wax biosynthesis were downregulated in BS, resulting in a decrease in the wax layer in BS. Genes related to lignin were upregulated at the transcriptional level, resulting in upregulation of metabolites related to phenylpropanoid biosynthesis. Expression of calcium-related genes were upregulated in BS. Cold-induced genes may represent the key genes that induce the formation of BS. In addition, the results demonstrated that exogenous NaH_2_PO_4_·2H_2_O and ABA treatment could inhibit the incidence of BS during harvest and storage time by increasing wax-related genes and calcium-related genes expression and increasing plant resistance, whereas the transcriptomics results indicated that GA_3_ may accelerate the incidence and index of BS.

**Conclusions:**

The results of this study indicate a molecular mechanism that could explain BS formation and elucidate the effects of different treatments on the incidence and molecular regulation of BS.

**Supplementary Information:**

The online version contains supplementary material available at 10.1186/s12870-021-03049-8.

## Background

Pear (*Pyrus spp.*), belongs to the subfamily Pomoideae in the family Rosaceae, the third most important temperate fruit species after grape and apple [1]. There are many pear varieties planted in China, and the main species cultivated for commercial production include sand pear (*P. pyrifolia* Nakai), ussurian pear (*P. ussuriensis* Maxim), white pear (*P. bretschneideri* Rehd), and Xinjiang pear (*P. sinkiangensis* Yu), as well as interspecific hybrid types [2].

‘Huangguan’ pear (*Pyrus bretschneideri* × *Pyrus pyrifolia*) is an early- and medium-maturing cultivar widely planted in northern China that has a high-quality and exquisite appearance after bagging [3]. This fruit has many excellent characteristics, such as a beautiful appearance, strong resistance, early fruit bearing and high yield in successive years, which are traits that are desired by the majority of producers and consumers. However, browning spot (BS) disease often occurs at the surface of ‘Huangguan’ pear fruits after bagging before harvest or during storage [4]. The symptoms of BS include an initial brown spot that spreads irregularly from the disease spot to the surroundings and becomes darker during fruit maturation [4, 5]. Whole fruit browning may occur in the later stages of this disease. Interestingly, this disorder affects only the exocarp of pear fruit, and the flesh and core are not affected [6]. Multiple lesions are connected into a round, irregular shape or chicken claw-like shape. Therefore, BS disorder is also known as chicken-claw disease by orchardman in China, and it causes a significant decrease in the commercial value of fruit for fruit farmers [7].

BS were first discovered in Xinji City, Hebei Province, in 1996. This disease mainly occurs on ‘Huangguan’ pears. However, a small number of green pear varieties, such as ‘Dangshansuli’ (*P. bretschneideri* Rehd.), ‘Lvbaoshi’ (*P. pyrifolia* Nakai), ‘Suisho’ (*P. pyrifolia* Nakai), ‘Xuehua’ (*P. bretschneideri* Rehd.) and ‘Xueqing’ (*P. pyrifolia* Nakai), also experience BS [8]. It was reported that BS disease of ‘Huangguan’ pear is an important physiological disorder [9–11] that mainly occurs in bagged fruits at the mature stage and after low-temperature storage [12–14]. In general, BS disorder of ‘Huangguan’ pear is affected by many factors, such as environmental factors (continuous rainfall and low temperature weather [12], chemical fertilizers use [15]), preharvest factors (bagging time, fruit bag type [5, 16], and swelling agent use [17]), and postharvest factors (cooling period duration [12–14, 18], storage temperature and CO_2_ and O_2_ concentrations [19–21]).

Some researchers believe that the thinning of the wax layer and skin cell wall of pears caused by bagging is the main cause of BS [16]. After bagging, the adaptability of fruit exocarp to severe environmental changes is reduced and the development of fruit exocarp is delayed. It has been reported that BS are closely related to calcium deficiency and phenolic dysregulation in pericarp tissue [9, 19, 22]. To date, research on BS disease has mainly focused on mineral nutrition (such as Ca [3, 5, 9, 11, 17, 23–27], Mg [5, 9], K [5, 9] and B [23]) and physiology and biochemistry [28, 29]. Additionally, the use of swelling agents may be another causes of BS [17]. Exogenous treatment with ethylene [4, 30], methyl jasmonate (MeJA) [31], 1-methylcyclopropene (1-MCP) [18] and CaCl_2_ [32] has been reported to affect the browning of postharvest ‘Huangguan’ pear. In addition, rapid postharvest cooling tends to increase BS formation, while slow cooling inhibits BS formation [12]. However, few studies have focused on the effect of exogenous phytohormone treatments, and the molecular mechanisms that regulate BS processes in ‘Huangguan’ pear.

This study observed the changes at the site of BS and analysed the molecular mechanism underlying BS formation at the transcriptomic and metabolomic levels. The incidence of BS after treatment with exogenous reagents [NaH_2_PO_4_·2H_2_O (P), abscisic acid (ABA), gibberellin A3 (GA_3_)] during harvest and storage was investigated. The key genes involved in exocarp formation were also analysed after treatment, which would provided a basis for the molecular mechanism underlying BS formation and elucidated the effect of different treatments on the molecular regulation of BS formation.

## Results

### Phenotype characteristics of BS disease of ‘Huangguan’ pear

**Fig. 1:**
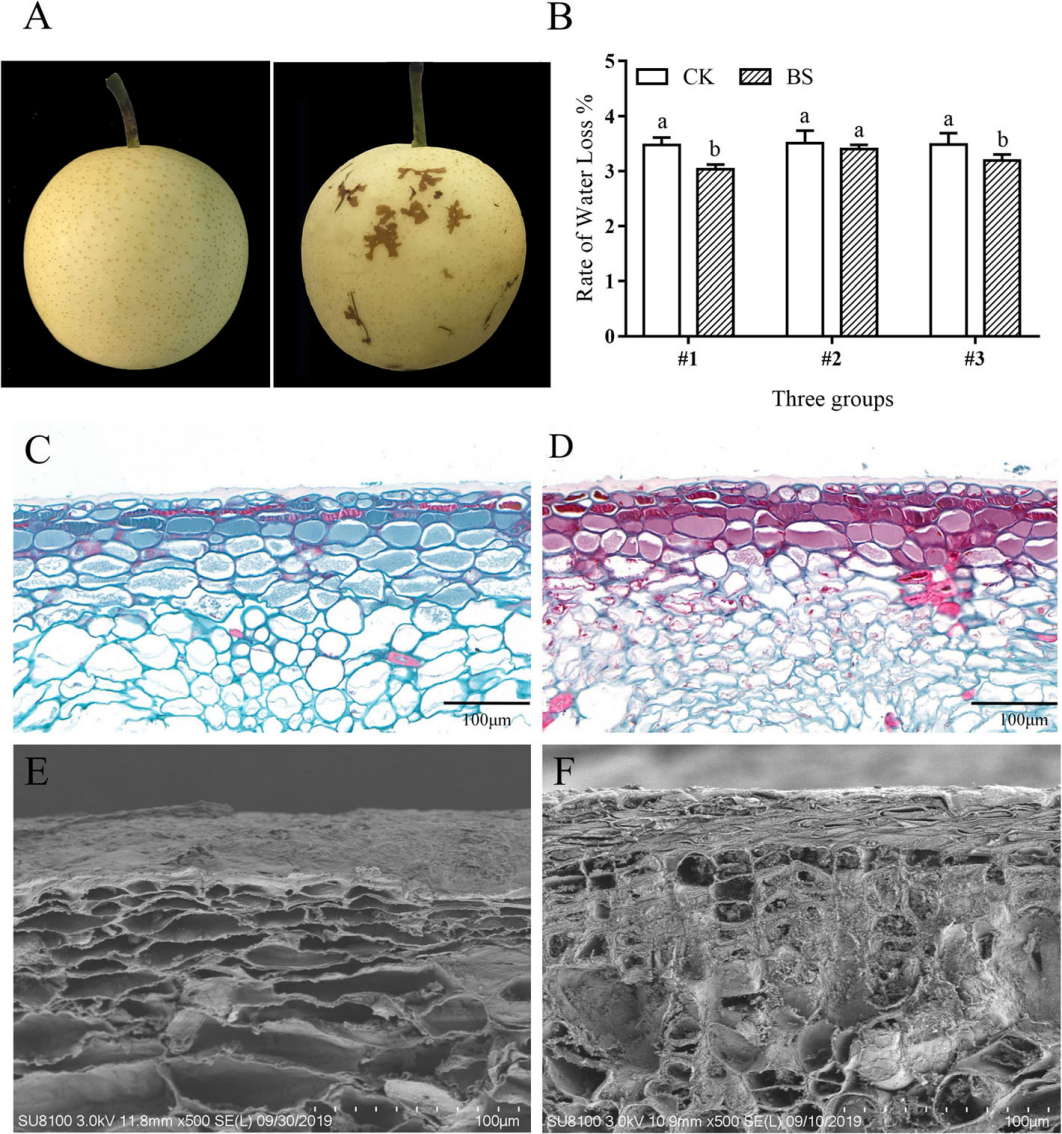
Phenotypic characteristics of BS of ‘Huangguan’ pear. (**A**) Phenotypes of normal ‘Huangguan’ pear and ‘Huangguan’ pear with BS disease. Observation of the paraffin sections of the normal part (**C**) and BS disease part (**D**) of ‘Huangguan pear’. SEM analysis of the normal part (**E**) and BS disease part (**F**) of ‘Huangguan’ pear. (**B**) RWL of CK and BS of ‘Huangguan’ pear at 10 days of storage under room conditions after harvest. The vertical bar indicates the standard error. The reported value is the mean ± SEM (*p* < 0.05). The ordinate represents three different groups, and each group has 10 fruits

Compared with the normal pear skin of ‘Huangguan’ pear, the BS-infected skin exhibits spots with an irregular, chicken claw-like shape that are randomly distributed over the surface (Fig. 1A). BS are a physiological disease that causes a slight depression in the affected area. Paraffin section observations revealed that the degree of lignification of the exocarp cells of the BS parts was significantly higher than that of the normal parts (Fig. 1C, D). SEM observations revealed a thick cuticular layer on the skin of the normal ‘Huangguan’ pear, however, the BS part of ‘Huangguan’ pear skin consisted of layers of dead cells and more densely arranged exocarp cells (Fig. 1 E, F). Those results indicated that the occurrence of BS may be caused by necrosis of the exocarp and hypodermal cortical tissues.

The SEM analysis also found that there were many cracks on the fruit surface, although the cracks on the BS part were covered by the lesions (Additional file 1: Fig. S1). Hence, an experiment to detect the rate of water loss (RWL) was conducted between CK and BS groups. After 10 d of storage at room temperature (25 °C), the RWL of the three groups of ‘Huangguan’ pear and ‘Huangguan’ pear with BS disease was calculated. The results showed that the RWL of groups #1 and #3 was significantly higher than that of ‘Huangguan’ pear with BS disease (Fig. 1B). The average RWL of the CK and BS groups was 3.49 and 3.19%, respectively. Therefore, BS lesions could inhibit water loss, which may be regulated by the layers of densely arranged dead cells at the fruit surface.

### Transcriptome and metabolome differences between the CK and BS fruits

RNA-Seq generated 6.24 gigabytes (GB) of clean data for each sample from the 5 complementary DNA (cDNA) libraries. A total of 30,596 expressed genes were identified, including 1212 new genes that were initially identified in this study. Successfully mapped reads ranged between 69.91 and 72.68%, and the average was 71.29% (Additional file 2: Table S1).

To compare the metabolites in the control group (CK) and BS-infected group (BS) metabolites of ‘Huangguan’ pears, datasets obtained from a Xevo G2 XS QTOF high-resolution tandem mass spectrometer (Waters) in electrospray ionization positive ion mode (ESI+) and negative ion mode (ESI−) were subjected to a principal component analysis (PCA). The results showed that metabolites from the CK and BS groups were clearly separated in the score plots, in which the first principal component (PC1) was plotted against the second principal component (PC2). (Additional file 1: Fig. S2 A, B). PLS-DA (plots from partial least squares discriminant) analyses were further performed to check the metabolite differences between the CK and BS groups (Additional file 1: Fig. S2 C, D), and the results showed significant biochemical differences between CK and BS.

**Fig. 2:**
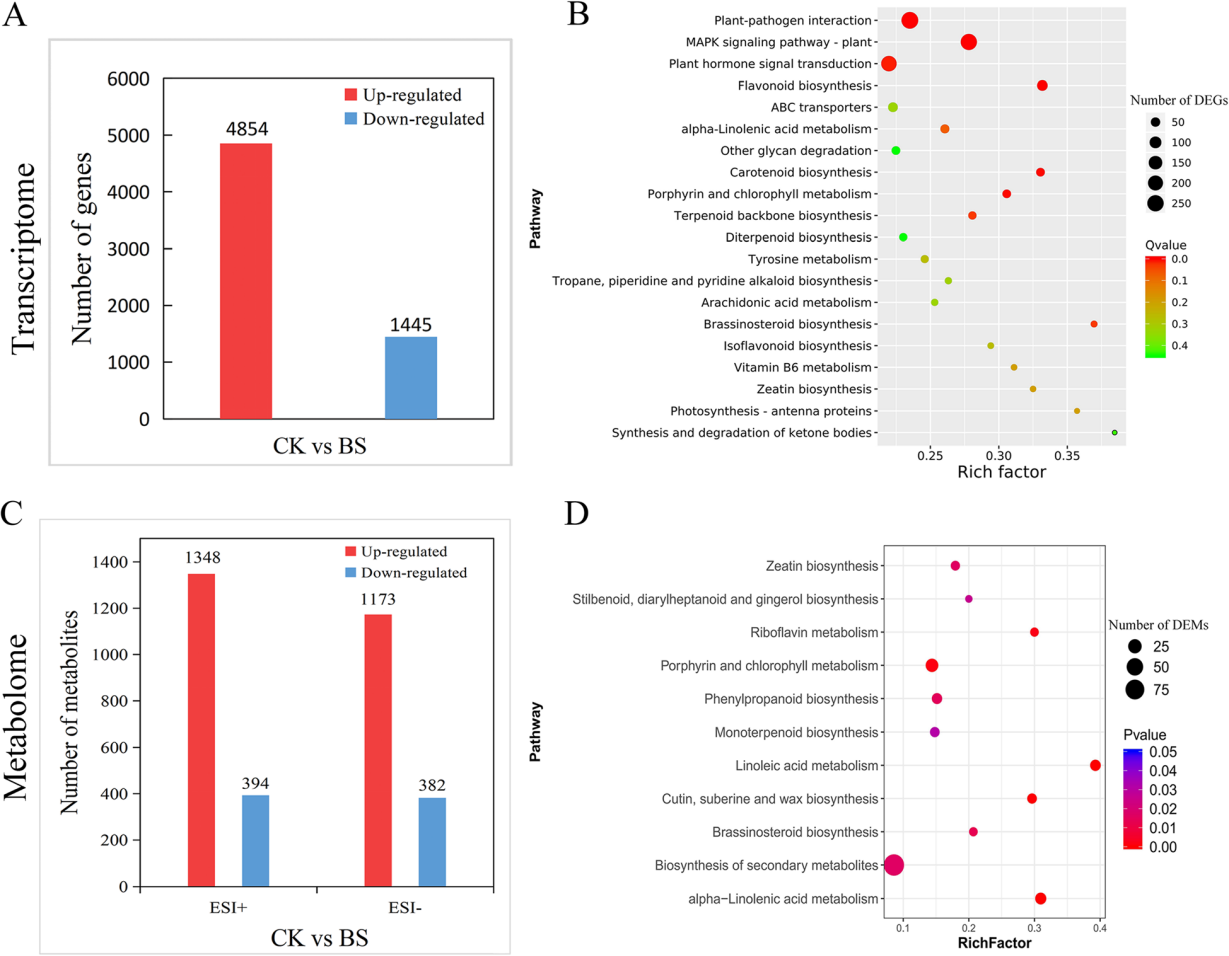
Significant DEG and DEM analysis between CK and BS. (**A**) Column chart of DEGs; red represents upregulated DEGs, and blue represents downregulated DEGs. (**B**) KEGG enrichment analysis of DEGs between CK and BS. The number of genes in each pathway is equal to the dot size. The dot color represents the q-value. The smaller the q-value, the redder the dot. (**C**) Numbers of upregulated (red) and downregulated (blue) metabolites. (**D**) KEGG enrichment analysis of differential metabolites. The number of DEMs in each pathway is equal to the dot size. The dot color represents the *P*-value. A redder point represents a smaller *P*-value

Transcript analyses of the two comparison groups by DESeq2 [33] identified 6299 DEGs, including 4854 upregulated and 1445 downregulated DEGs in the BS pear exocarp (Fig. 2A). To classify the functions of DEGs between the CK and BS groups, the assembled unigenes were annotated by using different protein databases (GO and KEGG) for homologous alignment. In the GO categories, DEGs were annotated with 1212 GO terms, with 1480 unigenes in biological process, 1906 unigenes in cellular component, and 1609 unigenes in molecular function, which included terms such as metabolic process, membrane and catalytic activity (Additional file 1: Fig. S3). KEGG pathway annotation analyses showed that the global and overview maps, carbohydrate metabolism, signal transduction and environmental adaptation were overrepresented (Additional file 1: Fig. S4). KEGG enrichment analyses were further performed to assess the DEGs between the CK and BS groups. We found seven significant pathways, including the MAPK signalling (245), flavonoid biosynthesis (72), plant-pathogen interaction (267), carotenoid biosynthesis (38), porphyrin and chlorophyll metabolism (37), plant hormone signal transduction (216) and brassinosteroid biosynthesis pathways (17) (Fig. 2B). To further identify the functions of BS-related genes, we analysed the gene expression in those significantly enriched pathways. The numbers of up- and downregulated genes are listed in Table 1.

**Table 1:** The top 7 enriched pathways of DEGs in BS

Pathway name	Type	Down	Pathway ID	Q-value
MAPK signaling pathway - plant	Environmental Information Processing	34	ko04016	4.86E-12
Flavonoid biosynthesis	Metabolism	21	ko00941	1.47E-06
Plant-pathogen interaction	Organismal Systems	22	ko04626	1.14E-05
Carotenoid biosynthesis	Metabolism	12	ko00906	0.001625608
Porphyrin and chlorophyll metabolism	Metabolism	13	ko00860	0.008369283
Plant hormone signal transduction	Environmental Information Processing	71	ko04075	0.008369283
Brassinosteroid biosynthesis	Metabolism	3	ko00905	0.02783563

These were selected with an FDR adjusted Q-value< 0.05

We characterized the exocarp of ‘Huangguan’ pear metabolomic changes in the BS disease parts. A total of 8829 and 8646 ions were identified in ESI+ and ESI− modes, respectively. After filtering low-quality ions that had RSD > 30%, 8432 and 7887 ions were retained in ESI+ and ESI− modes, respectively. Then, we identified differential metabolites between the CK and BS groups, and detected 1742 and 1555 differential ions in BS, including 1348 and 1173 upregulated ions and 394 and 382 downregulated ions in ESI+ and ESI− modes, respectively (Fig. 2C). In addition, 1581 and 781 differentiated metabolites were categorized into 96 and 74 KEGG pathways in ESI+ and ESI− mode, respectively (Additional file 3: Table S2). The KEGG enrichment analysis of differentiated metabolites (removing the duplicated ions in ESI+ and ESI− mode) showed that the biosynthesis of secondary metabolites, porphyrin and chlorophyll metabolism, cutin suberin and wax biosynthesis, phenylpropanoid biosynthesis, alpha-linolenic acid metabolism and brassinosteroid biosynthesis pathways were the most abundant (Fig. 2D). The differentially up- and downregulated metabolites are listed in Table 2.

**Table 2:** Enriched KEGG pathways of differential metabolites between CK and BS

Pathway	Count	Up	Down	Pathway ID
Biosynthesis of secondary metabolites	228	173	55	map01110
Phenylpropanoid biosynthesis	31	22	9	map00940
Porphyrin and chlorophyll metabolism	29	21	8	map00860
Flavonoid biosynthesis	18	16	2	map00941
Brassinosteroid biosynthesis	17	15	2	map00905
Carotenoid biosynthesis	16	13	3	map00906
Linoleic acid metabolism	16	14	2	map00591
alpha-Linolenic acid metabolism	13	12	1	map00592
Cutin, suberine and wax biosynthesis	11	11	0	map00073

### Analysis of DEGs and DEMs between the CK and BS groups

**Fig. 3:**
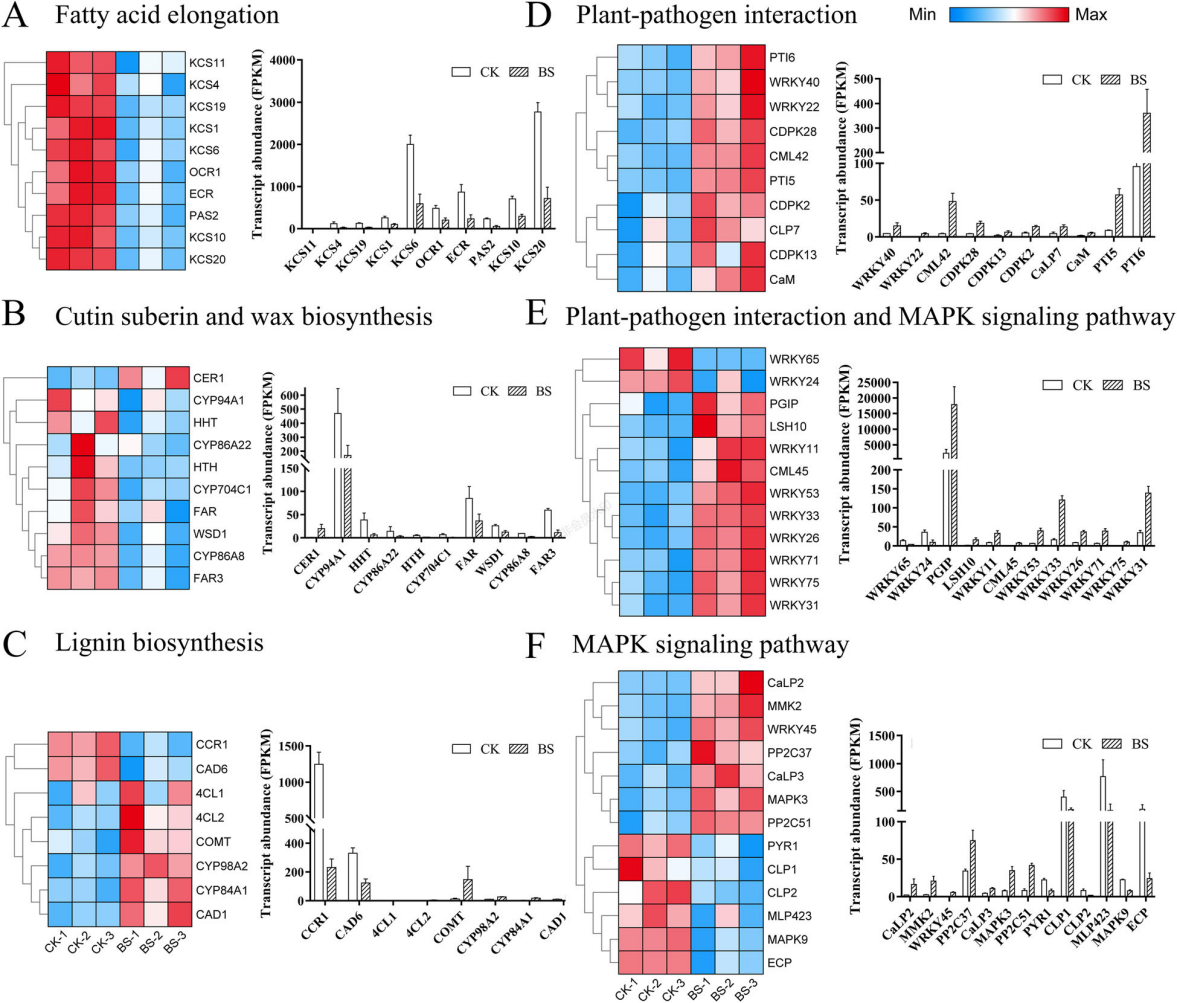
Significant DEGs between the CK and BS groups. Heatmap of DEGs involved in fatty acid elongation (**A**), cutin suberin and wax biosynthesis (**B**), lignin biosynthesis (**C**), plant-pathogen interaction (**D**), both plant-pathogen interaction and MAPK signalling pathways (**E**), and MAPK signalling pathway (**F**). Red represents upregulation, and blue represents downregulation

The phenotypic characteristics and metabonomics analysis of the pericarp indicated that the cutin suberin and wax biosynthesis pathway and lignin biosynthesis may be involved in the formation of BS. The fatty acid elongation pathway is upstream of cutin suberin and wax biosynthesis pathway [34]. At the transcriptome level, we found that many genes involved in wax biosynthesis were downregulated in BS, including the *CYP94A1*, *HHT*, *HTH*, *CYP704C1*, *WSD1*, and *FAR3* genes and 10 KCS family genes, indicating that the decrease in wax may be one of the causes of BS(Fig. 3A B). Six genes involved in lignin biosynthesis were upregulated, namely, *4CL2*, *CAD1*, *CYP84A1*, *4CL1*, *CYP98A2*, and *COMT1*, and two genes were downregulated, namely, *CAD6* and *CCR1* (Fig. 3C), resulting in the upregulation of metabolites in the phenylpropanoid biosynthesis pathway (Table 2). These results suggested that the formation of BS is caused by the decrease in epicuticular wax and the increase in lignified cells.

Transcriptome analysis revealed that plant-pathogen interactions (PPIs) and the MAPK signalling pathway (MAPK) are also key factors associated with BS (Fig. 3D-F). PGIPs (polygalacturonase inhibiting proteins) are associated with the plant cell wall and play a crucial role in plant defence [35], and they are upregulated in BS. LSH10 was a probable transcription regulator that acts as a developmental regulator by promoting cell growth in response to light, and it is upregulated in BS. We also detected six calcium-related genes differentially expressed in CK and BS*,* including *CaM*, *CML42*, *CML45*, *CaLP7*, *CaLP2*, and *CaLP3,* indicating that the calcium content in pear exocarp may be one of the factors affecting BS. In addition, we found 12 DEGs of the WRKY family in PPI and MAPK. These result indicated that the defence response caused by the change in calcium content in pear exocarp caused by bagging was involved in the formation of BS. Detailed information on these DEGs is listed in Additional file 4: Table S3.

**Fig. 4:**
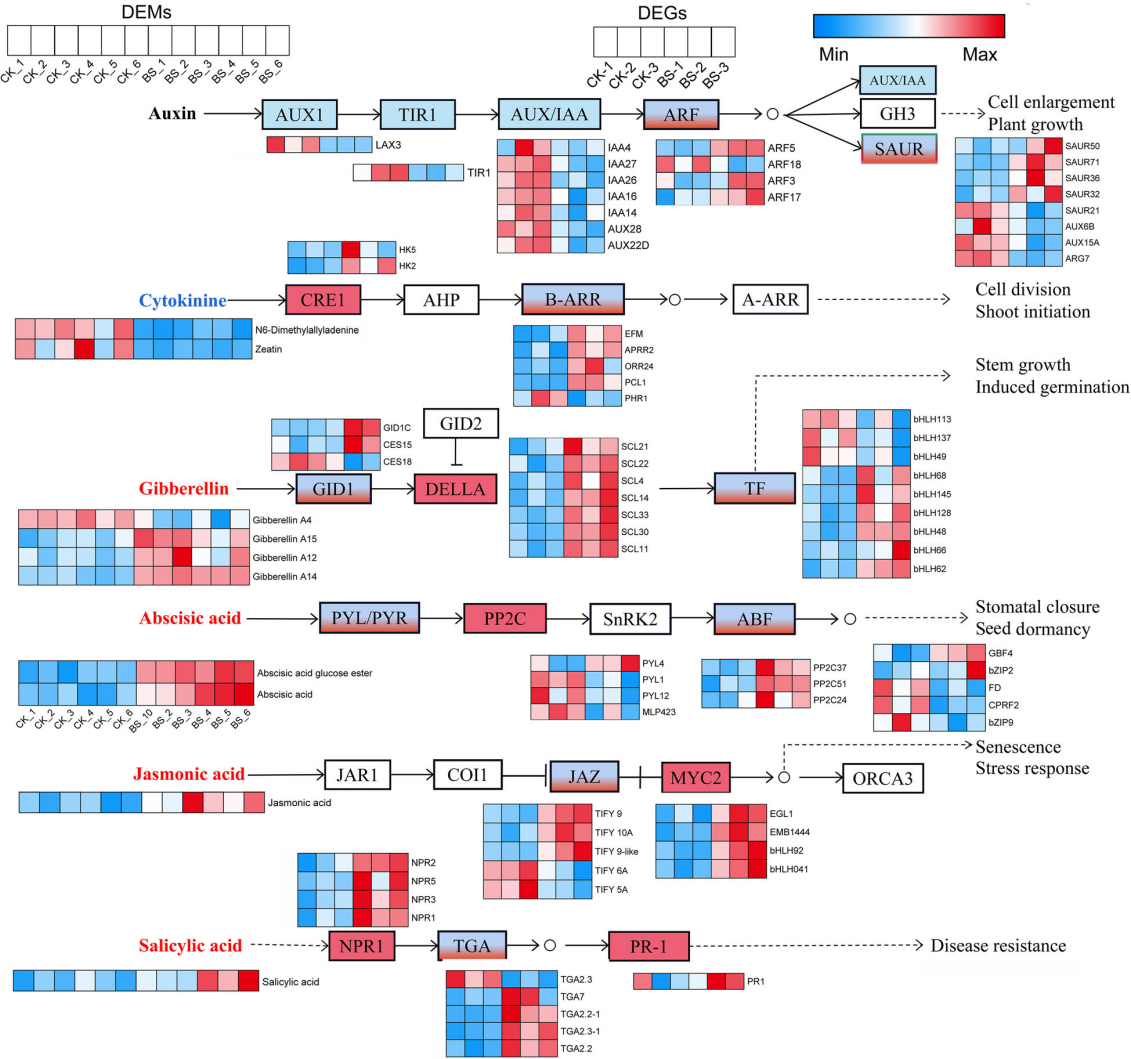
Significant DEGs and DEMs involved in plant hormone signal transduction between CK and BS. Red represents upregulation, and blue represents downregulation

Through metabolomics, we found that there was no significant difference in auxin (IAA) content between the CK and BS groups. Among cytokinins (CTKs), N6-dimethylallyladenine and zeatin were reduced in BS, while the content of gibberellins (GAs), abscisic acid (ABA), jasmonic acid (JA) and salicylic acid (SA) were all upregulated in BS except gibberellin A4 (Fig. 4). At the transcriptional level, we identified 216 DEGs involved in plant hormone signal transduction, including 55, 15, 37, 18, 23 and 12 DEGs in the IAA, CTK, GA, ABA, JA, and SA signalling pathways, respectively (Fig. 4). In the IAA signalling pathway, the *AUX1*, *TIR1*, and *AUX/IAA* genes were downregulated, which indicated that cell enlargement and plant growth were suppressed. In the CTK signalling pathway, the *CRE1* and *B-ARR* genes were upregulated, which indicated that cell division was promoted. In the GA signalling pathway, *GID1C* and *CES15* were upregulated among the *GID1* genes, and *SCL21*, *SCL22*, *SCL4*, *SCL14*, *SCL33*, *SCL30,* and *SCL11* were upregulated among the *DELLA* genes. Additionally, nine TFs in the bHLH family in the GA signalling pathway were identified, of which six were upregulated and three were downregulated. The increase in gibberellin content and expression of GA signalling genes in BS indicated that GA may have a certain promotion effect on BS. Greater ABA, JA and SA content in BS was observed in the BS group, and the gene expression of ABA, JA and SA signalling pathways was also significantly increased, which induced the disease resistance in the plants. Detailed information on all genes involved in plant hormone signal transduction is listed in Additional file 4: Table S4.

**Fig. 5:**
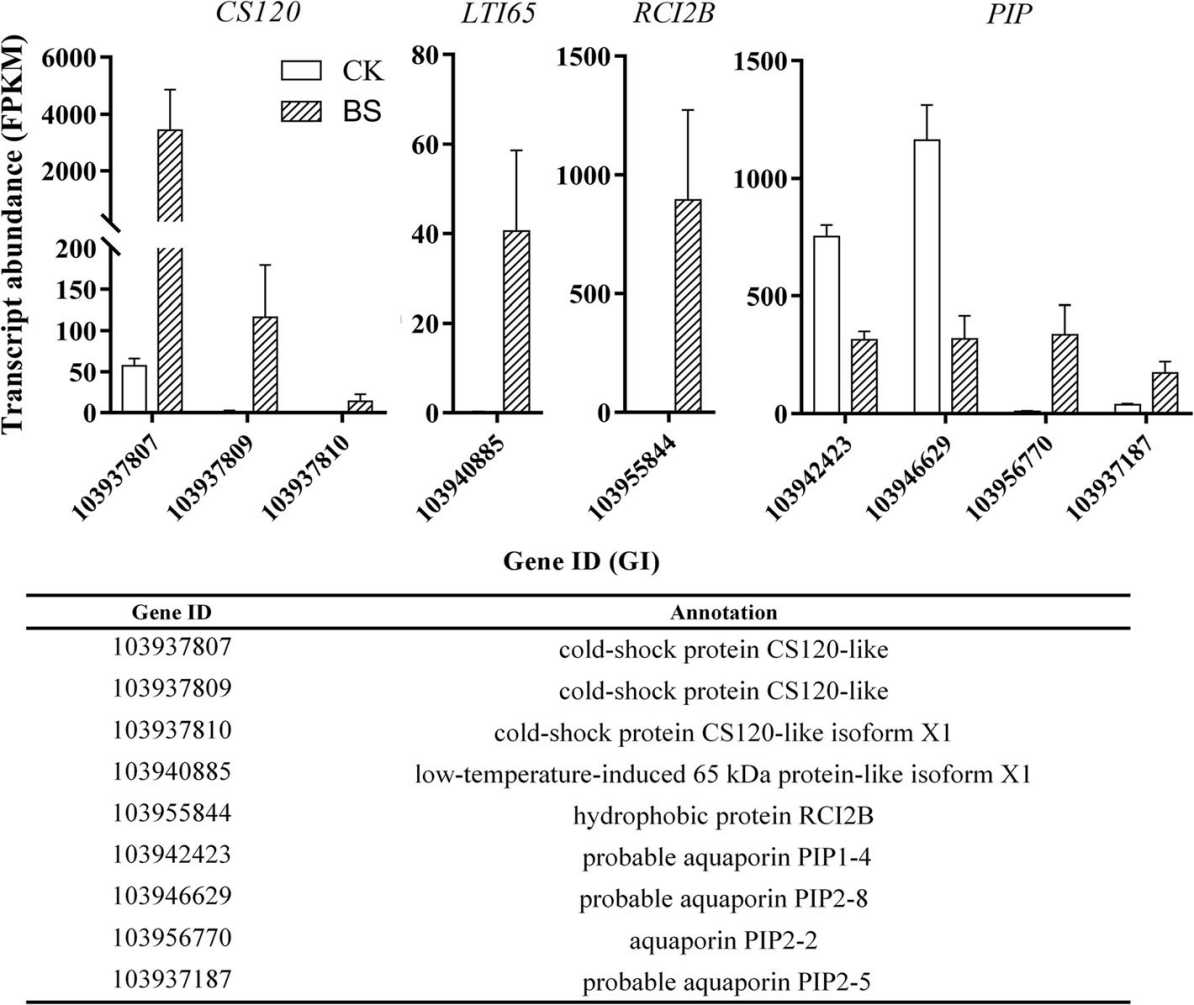
Transcript abundance of significant DEGs between CK and BS. The error bars are the means ± SEM of three biological repeats

BS has been reported to be associated with a sudden drop in temperature. Cold exercise or slow cooling are commonly used in production to reduce the incidence of BS [12–14]. We identified three cold-shock protein CS120-like (*CS120*) genes (gene ID:103937809, 103,937,810, 103,937,807) and one low-temperature-induced 65 kDa protein-like isoform X1 (*LTI65,* gene ID: 103940885) that were significantly upregulated in BS (Fig. 5). Hydrophobic protein RCI2B (*RCI2B,* gene ID: 103955844) has been proven to be a cold-induced gene [36] that is upregulated in BS. Aquaporin is a membrane protein that was originally characterized as a water channel through which H_2_O could permeate biological membranes [37]. Four DEGs in aquaporin PIP (gene ID: 103946629, 103,942,423, 103,937,187, 103,956,770), *PIP1–4* and *PIP2–8* were upregulated, while *PIP2–2* and *PIP2–5* were downregulated in BS.

### Transcription factors (TFs) involved in BS formation

TFs are important regulators that activate or repress the expression of both coding and noncoding genes to influence or control many biological processes [38]. In our analysis of the transcriptome data, we detected 423 differentially expressed TFs between the CK and BS groups, including 341 upregulated and 82 downregulated TFs. The AP2-EREBP, MYB and WRKY families were the most abundant TF families between the CK and BS groups, followed by the bHLH, NAC, C2H2, and HSF families (Table 3).

**Table 3:** Differentially expressed transcription factors (TFs) between CK and BS

TF family	Number	Up	Down	Description
AP2-EREBP	53	40	13	Ethylene-responsive transcription factor
MYB	49	43	6	MYB-related protein
WRKY	46	44	2	WRKY DNA -binding domain
bHLH	35	21	14	Helix-loop-helix DNA-binding domain
NAC	26	24	2	NAC domain-containing protein
C2H2	17	16	1	Zinc finger protein
HSF	16	16	0	Heat stress transcription factor
GRAS	15	15	0	scarecrow-like protein
LOB	13	10	3	LOB domain-containing protein
MADS	10	8	2	SRF-type transcription factor
G2-like	10	9	1	myb-related protein
C3H	9	8	1	Zinc finger CCCH domain-containing protein
mTERF	9	8	1	mTERF domain-containing protein
C2C2-Dof	9	6	3	dof zinc finger protein
TCP	7	5	2	Circadian rhythm - plant
FHA	7	5	2	FHA domain-containing protein
C2C2-GATA	7	4	3	GATA-binding protein
Tify	7	5	2	jasmonate ZIM domain-containing protein
C2C2-CO-like	7	6	1	zinc finger protein CONSTANS
ABI3VP1	6	3	3	B3 domain-containing protein
Trihelix	6	5	1	trihelix transcription factor
OFP	6	3	3	isoleucyl-tRNA synthetase
FAR1	5	5	0	zinc finger SWIM domain-containing protein
ARF	5	3	2	auxin response factor
other TFs	43	29	14	
total	423	341	82	

### Coexpression network of BS-related genes

**Fig. 6:**
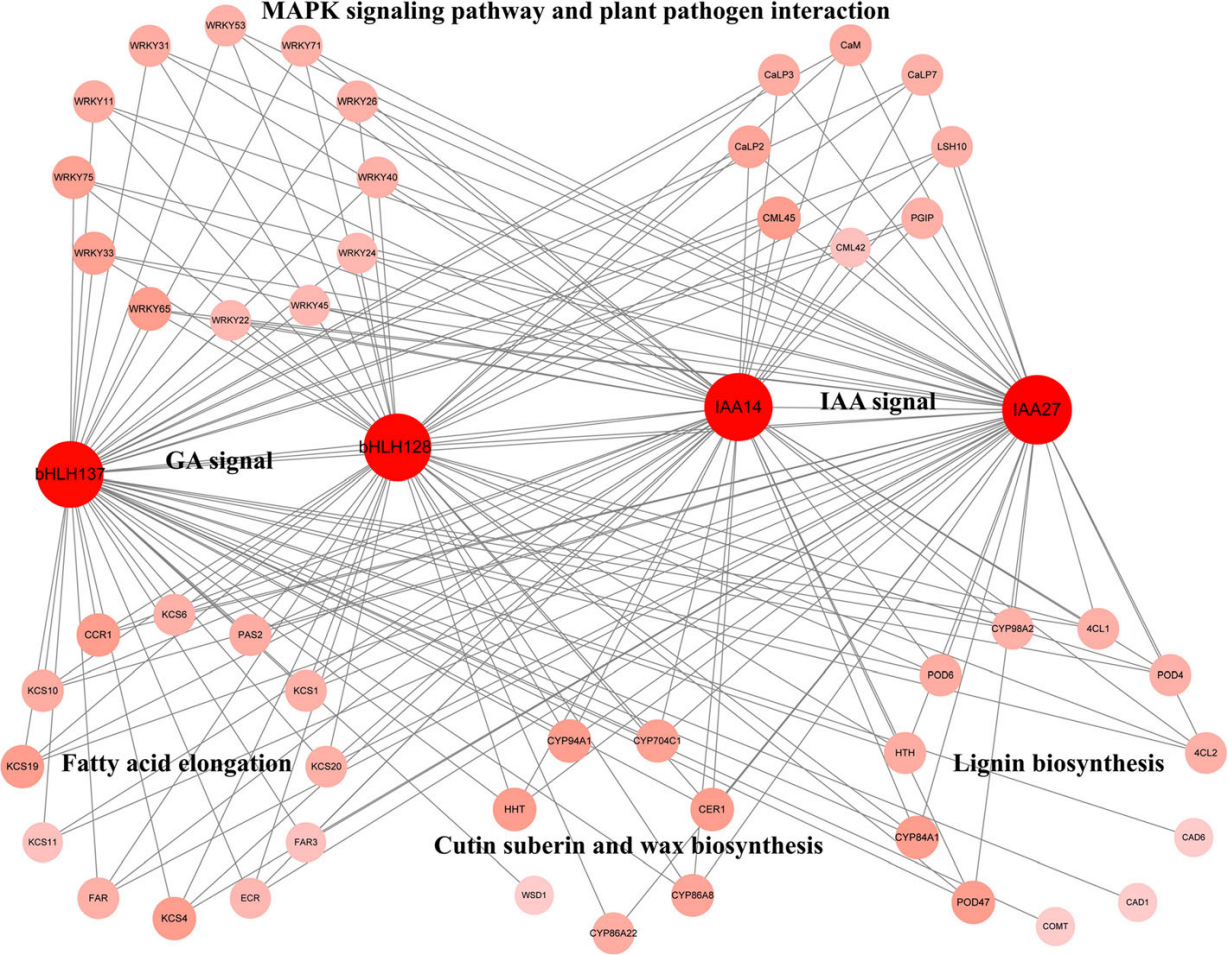
Coexpression network of genes involved in BS formation. Detailed information on the genes is listed in Additional file 4: Table S3 and Table S4

In our transcriptome analysis, we found that wax, lignin, calcium, plant hormone signal transduction, and cold-induced genes were the key genes for BS formation. We performed a coexpression network analysis to illuminate the collaboration between those genes, and the analyses with transcriptome data showed that GA signal and IAA signal genes were classified into different coexpression clusters with wax, lignin biosynthesis and calcium-related genes (Fig. 6). We found that *bHLH137*, *bHLH128*, *IAA14* and *IAA27* were coexpressed with multiple genes involved in fatty acid elongation, cutin, suberin and wax biosynthesis, lignin biosynthesis, MAPK and PPI. These findings indicate that the formation of BS may be regulated by plant hormone signals, especially IAA and GA signals.

### Combined analysis of the metabolome and transcriptome

**Fig. 7:**
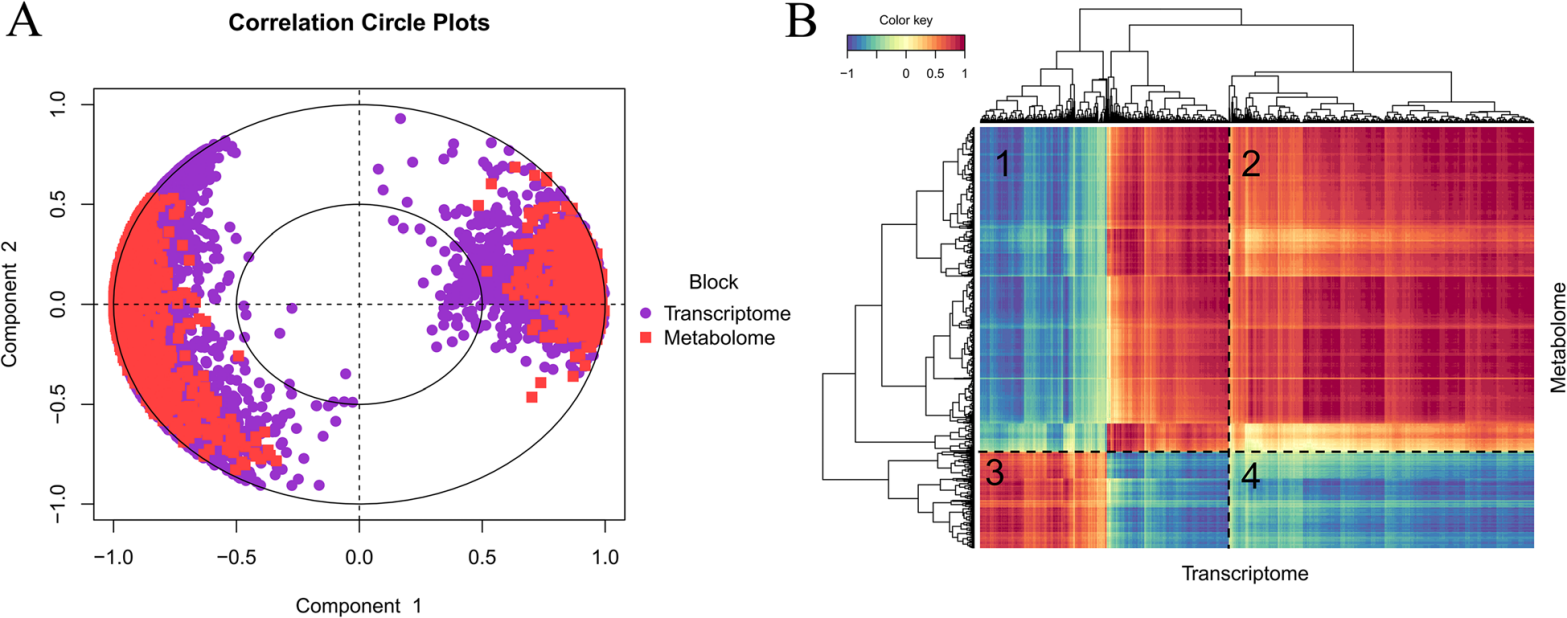
Combined analysis of the metabolome and transcriptome between the CK and BS groups. (**A**) Concentric diagram of the correlation of DEGs and DEMs between the CK and BS groups. Each point in the circle represents a gene, and each square represents a metabolite. If the angle between the DEG and DEM is an acute angle, then the correlation is positive. If the angle is the deltoid angle, then the correlation is negative. In general, variables far away from the centre of the circle are more closely related. (**B**) Heatmap cluster of DEGs and DEMs. Each row represents a DEM, and each column represents a DEG. Blue represents a negative correlation, and red represents a positive correlation

MixOmics [33] multifunctions were used for multivariable dimensionality reduction to explore the relationship between transcriptomics and metabolomics (Fig. 7A). The block splsda function in mixOmics was used to analyze differential genes and differential metabolites, and the plotVar and circosPlot functions were used to visualize the results. We found that DEGs and DEMs were closely correlated. In general, most DEGs and DEMs are far from the center of the circle, which means a strong correlation between them. Alternatively, a regularized canonical correlation analysis (rCCA) [39] was performed to measure the degree of correlation between genes and metabolites (Fig. 7B). In total, a correlation between 6299 DEGs and 1280 DEMs was detected. We named the four quadrants with numbers 1–4. The results showed that quadrants 1 and 4 represent the opposite expression trend of DEGs and DEMs, suggesting that the expression of DEGs and DEMs had a negative correlation. In contrast, quadrants 2 and 3 represent the consistent expression trend of DEGs and DEMs, indicating that those genes may be a positively regulated by metabolites.

### Effects of P, ABA and GA_3_ treatments on BS

**Fig. 8:**
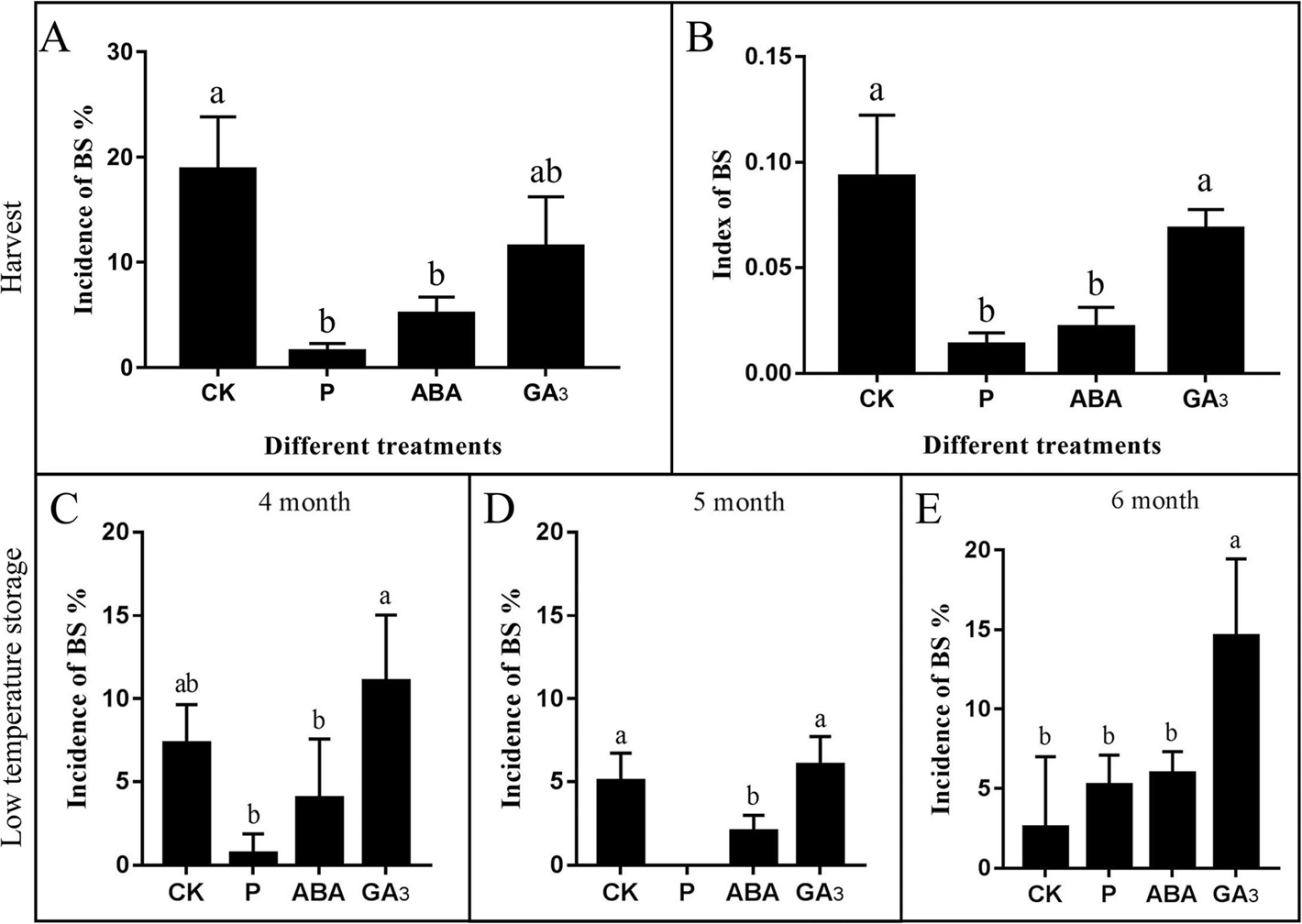
The incidence of BS disorder after different treatments in ‘Huangguan’ pears. (**A**) Incidence of BS disorder treated with exogenous P, ABA, and GA_3_. (**B**) Index of BS disorder treated with exogenous P, ABA, and GA_3_. Incidence of BS disorder with different treatments after 4 (**C**), 5 (**D**), and 6 (**E**) months of storage. The error bars are the means ± SEM of three biological repeats. (*P* ≤ 0.05)

Treatments with P, ABA, and GA_3_ were performed to investigate their effects on the BS of ‘Huangguan’ pear (Fig. 8A, B). The P and ABA treatments significantly reduced the incidence and index of BS. The incidence and index of BS treated with GA_3_ were higher than those of the other treatments. The results showed that the P treatment had the best inhibitory effect on BS disorder, and ABA treatment also had a certain inhibitory effect on BS, and the GA_3_ treatment promoted the occurrence of BS.

In addition, we investigated the BS incidence of ‘Huangguan’ pears with different treatments during storage (Fig. 8C, D, E). We found that the P treatment effectively inhibited BS at 4 and 5 months of storage (Fig. 8C, D), while the ABA treatment inhibited BS at 5 months of storage, but the result did not significant differ from that of the other time periods compared with the CK (Fig. 8D). The incidence of BS was higher after GA_3_ treatment during storage, indicating that GA_3_ may promote BS after low-temperature storage (Fig. 8C, D, E).

### Transcriptomics analysis of pear exocarp after P, ABA, and GA_3_ treatments

**Fig. 9:**
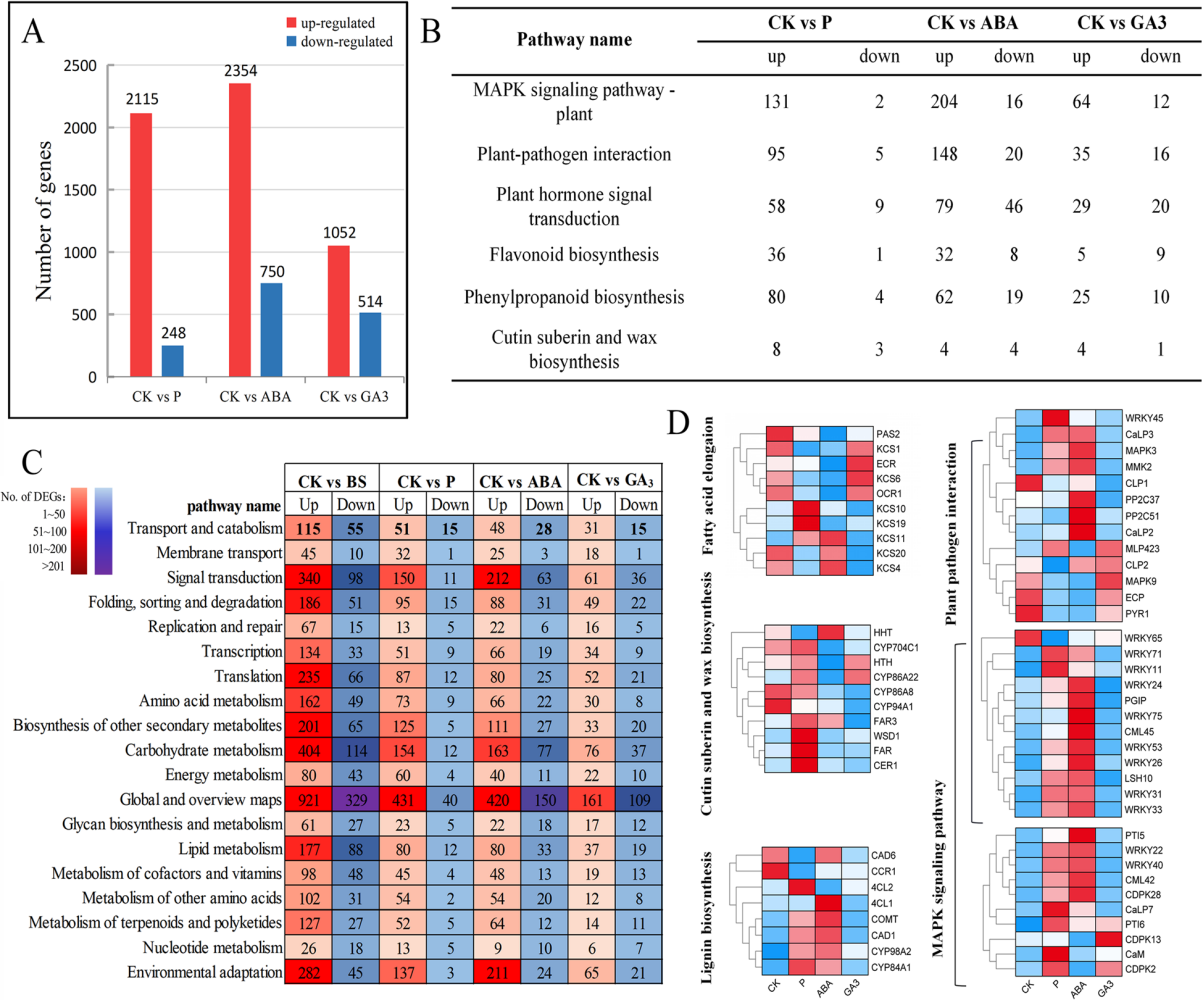
Significant DEGs between the CK^_^BS, CK-P, CK-ABA, and CK-GA_3_ comparison groups. (**A**) Column chart of the number of DEGs. (**B**) The up- and downregulation of genes involved in BS-related pathways after different treatments. (**C**) KEGG annotation of DEGs. (**D**) Expression of genes involved in BS related pathway after treatments. Red represents upregulation, and blue represents downregulation

We analysed the changes at the transcription level of pear exocarp to explore the effects of different treatments on the occurrence of BS. After the P treatment, 2363 DEGs were identified, including 2115 upregulated genes and 248 downregulated genes. A total of 3104 DEGs occurred after treatment with ABA, including 2354 upregulated genes and 750 downregulated genes. The GA_3_ treatment caused 1566 DEGs, including 1052 upregulated genes and 514 downregulated genes (Fig. 9A). To classify the functions of DEGs after different treatments, KEGG annotation analysis was carried out, and it showed that the global and overview maps, carbohydrate metabolism, signal transduction and environmental adaptation were overrepresented (Fig. 9C). Furthermore, we identified the expression of genes involved in BS formation (Fig. 9B) and found that wax biosynthesis-related genes, such as *KCS10*, *KCS19*, *KCS11*, *FAR3*, *WSD1*, *CER1*, were upregulation after P treatment. Similarly, ABA treatment also increased the expression of wax-related genes, including *KCS11*, *KCS20*, *KCS4*, *FAR3*. Moreover, treatment with P and ABA increased the expression of many genes involved in the PPI and MAPK pathways, including calcium-related genes (*CaM*, *CaLP3*, *CaLP2*, *CaLP7*, *CML42*, and *CML45*) and WRKY TFs (*WRKY71*, *WRKY11*, *WRKY24*, *WRKY75*, *WRKY53*, *WRKY26*, *WRKY22*, and *WRKY40*), which may improve the plant’s resistance to disease. However, the effect of GA_3_ treatment was not obvious. These results are consistent with the previous incidence of BS observed after the three treatments.

### Gene expression analysis by q-RT-PCR after treatment

**Fig. 10:**
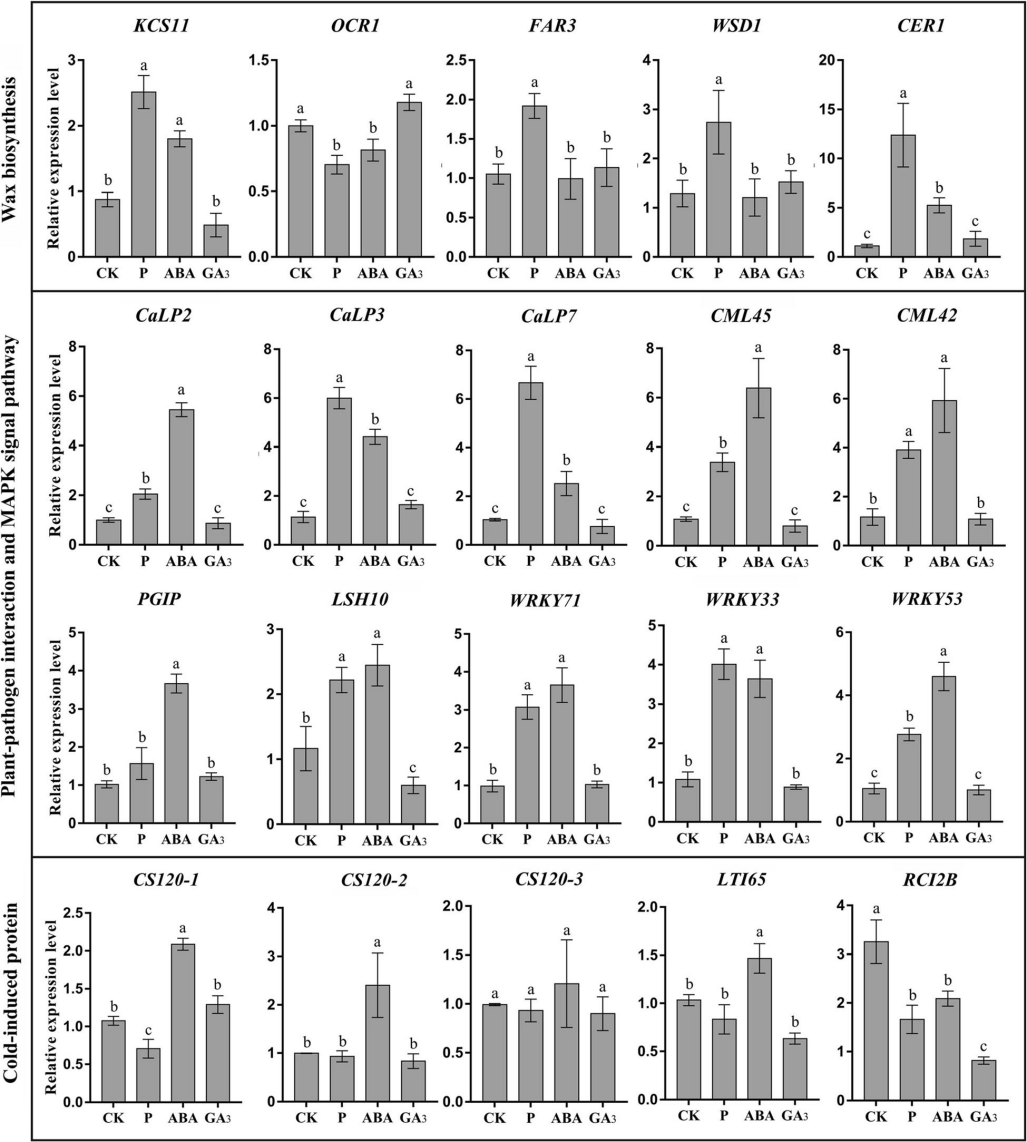
q-RT-PCR verification of genes related to BS after different treatments. The error bars are the means ± SEM of three biological repeats (*P* ≤ 0.01)

Previous studies have shown that a reduction of the wax layer may be one of the causes of BS. Therefore, we analysed the expression of five wax-related genes in the pericarp of ‘Huangguan’ pear after different treatments (Fig. 10). We found that *KCS11*, *FAR3*, *WSD1*, and *CER1* were upregulated after the P treatment and *KCS11* and *CER1* were upregulated after the ABA treatment. The expression of *OCR1* was downregulated after the P and ABA treatments but did not significantly differ after the GA_3_ treatment. BS has been reported to be related to calcium deficiency in the peel [3, 9, 22]. Five calcium-related genes, *CaLP2*, *CaLP3*, *CaLP7*, *CML45*, and *CML42*, were upregulated after the P and ABA treatments but did not show significant changes in expression after the GA3 treatment (Fig. 10). Additionally, five genes involved in both PPI and MAPK can be activated by various biological and abiotic stresses [13], including *PGIP* and *LSH10* and the three WRKY family TFs *WRKY53*, *WRKY71*, *WRKY33*. Among them, the expression of *LSH10, WRKY53, WRKY71,* and *WRKY33* increased to different degrees after the P and ABA treatments. The expression of *PGIP* was increased after ABA treatment. However, GA_3_ treatment did not affect the expression of these genes and even had a persistent effect (Fig. 10). These results are consistent with the transcriptome data.

Furthermore, the expression of five cold-induced genes was detected, namely, *CS120–1* (gene ID: 103937807), *CS120–2* (gene ID: 103937809), *CS120–3* (gene ID: 103937810), *LTI65* and *RCI2B.* The results show that the ABA treatment increased the expression of *CS120–1*, *CS120–2* and *LTI65,* while *CS120–1* and *LTI65* were downregulated after the P treatment. The expression of *RCI2B* was decreased after all tree treatments. The results show that ABA treatment may improve the adaptability of fruit to chilling injury, while the effect of P and ABA treatment on the expression of cold-related genes was not obvious.

### Regulatory network of BS formation

**Fig. 11:**
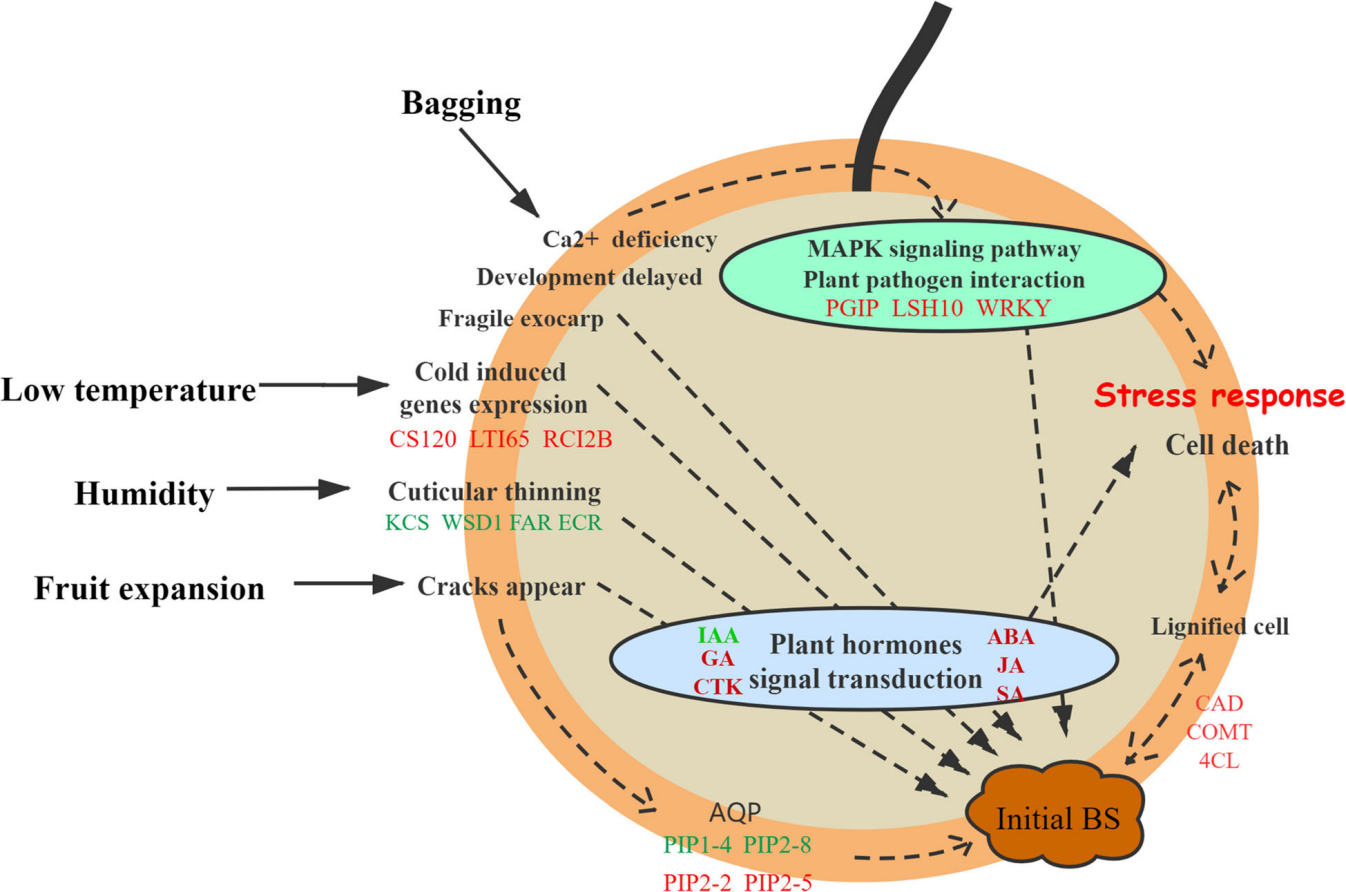
A proposed model of BS formation in ‘Huangguan’ pear fruits. The red color represents upregulation and green color represents downregulation. The detailed gene information can be viewed in Additional file 4: Table S3, Table S4 and Fig. 6. IAA, Auxin; GA, gibberellic acid; CTK, cytokinin; ABA, abscisic acid; JA, jasmonic acid; SA, Salicylic acid

According to our investigation and research, we believe that many factors result in BS, especially the low temperatures. The possible regulatory network is shown in Fig. 11. The development of fruit exocarp is delayed, and the concentration of Ca^2+^ is reduced after bagging. The fragile peel cannot withstand swelling when the fruit enlarges. When the temperature drops, the peel is stretched, cracks appear, and low temperature-induced genes are upregulated, which causes a series of defensive reactions through the PPI and MAPK pathways. In addition, the high humidity conditions in bags cause cuticular thinning of the pear exocarp, which may cause cracks on the fruit surface. Then, dead cells accumulate near those cracks, which ultimately become BS.

## Discussion

### Factors influencing BS on ‘Huangguan’ pear

BS disease is the main disease of ‘Huangguan’ pear and primarily occurs in bagged fruits at the mature stage. However, a small proportion of BS cases has also been found on unbagged fruits, although the shape of the disease is mostly circular and not consistent with that of bagging (Additional file 1: Fig. S5). Therefore, bagging may not be the only cause of BS. We observed that the onset of BS was characterized by a close arrangement of lignified dead cells accompanied by a significant reduction in epidermal wax (Fig. 1). The transcriptomic analysis showed that the expression of wax-related genes in BS was decreased while the expression of lignin-related genes was increased (Fig. 3), which was consistent with the observed phenotypic phenomenon. However, the cause of this phenomenon is still unclear.

It has been reported that BS is associated with sudden decreases in temperatures [12, 13, 18]. BS has been considered a chilling injury symptom in cold-stored ‘Huangguan’ pear [30]. Studies have shown that ‘Huangguan’ pear is susceptible to BS disorder a few days after low-temperature storage [4]. It has been reported that MeJA can improve the chilling resistance of eggplant (*Solanum melongena* L.), and also can inhibit browning disorder [31, 40, 41]. This finding indicated that BS may be caused by low temperature. We detected four low temperature-induced genes by transcriptomics that were highly expressed in BS but barely expressed in the normal pericarp, including the *CS120*, *LTI65* and *RCI2B* genes (Fig. 5). Protein synthesis is generally inhibited when the temperature drops abruptly, and the production is significantly lower than at a normal physiological temperature; however, cold-shock proteins (CSPs) increase dramatically under these conditions [42]. *LTI65* and *RCI2B* are induced by low temperature in *Arabidopsis thaliana* [36, 43]. Li. et al. [13] studied the effect of cold exercise treatment on ‘Huangguan’ pear, and the results showed that cold exercise effectively inhibited fruit peel brown spots and had no obvious effect on storage quality. Based on these findings, low temperature is indeed one of the causes of BS.

Calcium deficiency in the pericarp is also responsible for BS [3, 5, 9, 11, 17, 23–27]. Studies have shown that the water-soluble and total Ca^2+^ contents in both the skin and flesh tissue and the total Ca^2+^ content only in the skin of fruits with BS were significantly lower than those of fruits without BS [3]. Alternatively, stress can not only induce calcium signalling but also the expression of calcium-binding proteins in plants [44]. Ferguson suggested that an imbalance in Ca^2+^ contents leads to metabolic disorders that result in physiological diseases [45]. In our study, the expression of calcium-related genes in infected and unaffected pericarp was analysed via transcriptomic. We detected six calcium-related genes that were upregulated in BS, namely, *CaLP2*, *CaLP3*, *CML45*, *CML42*, *CaLP7*, and *CaM*. These genes are involved in the PPI and MAPK pathways. In addition, studies have shown a close relationship between Ca^2+^ and aquaporin (AQP) activity [46]. The effect of Ca^2+^ on AQP activity is mainly achieved through CDPK [47]. Certain environmental factors, such as drought, low temperature, light exposure and nutritional deficiency, can promote the expression of the AQP gene [48, 49]. We detected four AQP genes that showed differential expression, namely, *PIP1–4*, *PIP2–8*, *PIP2–2*, and *PIP2–5* (Fig. 5). The AQP genes may affect BS by regulating the calcium concentration.

The MAPK signalling pathway was the most significantly enriched pathway in the CK-BS comparison group, and it is associated with various physiological, developmental and hormonal responses [50]. Molecular and biochemical studies have revealed that MAPK activation correlates with stimulatory treatments, such as low temperature, drought, wounding, pathogen infection, hyper and hypo-osmolarity, and reactive oxygen species [51–55]. Genes involved in both the PPI and MAPK pathways have been detected. *PGIP* was proven to changes the composition of the degradation products in the cell wall of pear fruit and increases the content of pectin monomer to induce the disease resistance of plants [56], which was upregulated in BS. WRKY family TFs are involved in the plant defence response [57]. We detected 12 WRKY family TFs that showed differential expression (Fig. 3). Therefore, BS disease may be a manifestation of fruit responses to adverse environments.

Plant hormone signal transduction also plays a critical role in the formation of BS. Hormonal cues regulate many aspects of plant growth and development, thereby facilitating the ability of plants to respond to environmental changes systemically [58]. We found that genes involved in the IAA signalling pathways were downregulated, while genes involved in the GA and CTK signalling pathways were upregulated (Fig. 4). Cold temperatures have been shown to inhibit plant growth by reducing auxin accumulation [59]. Alternatively, a previous study showed that low temperature induces an increase in GA_3_ sensitivity [60]. We predict that low temperature causes the differential expression of plant hormone signalling pathway genes, which indicates that low temperature might be the most important cause of BS.

Furthermore, the humidity in fruit bags may be another factor that affects BS. Studies have shown that wax is influenced by temperature, light intensity and humidity [61], and that high humidity inhibits wax synthesis [62]. In addition to wax, there are reticular or strip cracks on the fruit surface caused by the continuous expansion of flesh cells during the development stage, thus leading to epidermal expansion and cracking. Some studies have found that these cracks are easily affected by external environmental factors [63]. These cracks may be the cause of BS. Under the action of AQP, brown spots are formed in pear fruits. Therefore, humidity may be a critical impact factor on BS formation.

### Effects of different treatments on BS of ‘Huangguan’ pear

Key differentially expressed genes in BS were screened by transcriptome analysis. The different treatments showed that P and ABA significantly inhibited the incidence of BS. Then, the expression of key genes at the transcriptional level after the treatments was analysed. The results showed that P treatment could improve the expression of the wax-related genes *WSD1* and *FAR*, resulting in a thicker cuticle. The expression of calcium-related genes *CaLP3*, *CML45*, *CML42*, *CaLP7*, and *CaM* was upregulated, which could alleviate calcium deficiency in the fruit exocarp. Additionally, P treatment improved the expression of genes involved in both the PPI and MAPK pathways, including *LSH10*, *WRKY53*, *WRKY71*, *WRKY33*, *WRKY31*, *WRKY26*, and *WRKY11*, which improved the adaptability of fruit to adverse environments, thereby inhibiting the incidence of BS.

ABA treatment also had a certain inhibitory effect on BS. ABA has been reported to control the expression of wax synthesis genes and prevent leaf water loss [64]. However, it is a major hormone involved in the plant response to stress. In our results, we found that ABA treatment can increase the expression of the calcium-related genes *CaLP2*, *CaLP3*, *CML45*, *CML42*, and *CaLP7* and the adaptability of fruits might be improved by increasing the expression of *PGIP*, *LSH10*, *WRKY53*, *WRKY71*, *WRKY75*, *WRKY33*, *WRKY31*, *WRKY26*, *WRKY24*, and *WRKY11*. In general, ABA treatment may roughen the exocarp and improve the disease resistance of the fruit.

## Conclusion

This study shows that the occurrence of BS was accompanied by a reduction in the wax layer and the accumulation of dead cells via lignification. At the transcription level, genes related to wax synthesis were greatly downregulated, genes related to suberin and lignin biosynthesis were greatly upregulated, and genes related to calcium and low temperature were upregulated. In addition, the endogenous hormone content between the CK and BS groups differed based on a decrease in CTK and an increase in ABA, JA, GA and SA, and these findings were consistent with the expression trend of their signal transduction-related genes except for CTK. We also found that the P and ABA treatments inhibited BS to varying degrees while the GA_3_ treatment may promote BS. The expression levels of key genes involved in BS formation after the different treatments were consistent with the morbidity results. These results provide a theoretical basis for the molecular mechanism underlying ‘Huangguan’ pear browning spot disease.

## Methods

### Plant materials and treatment

Ripe ‘Huanguan’ pears (CK) and ‘Huangguan’ pears with BS disorder (BS) were harvested from an orchard in a gardening field of Dangshan County, Suzhou City, Anhui Province, during the harvest season in 2018. Treatments were carried out by spraying NaH_2_PO_4_·2H_2_O (0.2%, Sigma 04269), ABA (100 μM, Sigma A1049), and GA_3_ (300 mg/L, Sigma G8040) on ‘Huangguan’ pears at 10, 20, and 30 days after full bloom (DAFB). Reagent treatments are commonly used in fruit bags during production. Each treatment had three biological replicates, and each tree had approximately 120 treated fruits.

Pears were immediately transported to the laboratory at Anhui Agricultural University (Hefei, China) after harvest. The 0.5 mm thickness exocarp was dissected from the fruit skin with a double-sided blade. Six biological replicates for metabolic profiling were collected randomly from the CK and BS of ‘Huangguan’ pear exocarp. Three biological replicates of the CK, BS and different hormone treatments were used for RNA sequencing (RNA-Seq). The collected fruit samples were frozen in liquid nitrogen immediately and then stored at − 80 °C.

### Observation of paraffin sections and scanning electron microscopy of pear exocarp

After removing the dirt on the fruit surface, a 0.6 cm × 0.7 cm piece was cut on the pear surface with a double-sided blade and immediately fixed in FAA solution. A 3 mm tissue block was cut with a sharp blade and then fixed in electron microscope fixing solution. The preparation of the fruit for paraffin sections and electron microscopy were conducted at Servicebio (WuHan) Biotechnology Co., Ltd.

### Pear postharvest water loss measurement

‘Huangguan’ pear fruits with BS disease of the same size were stored at room temperature conditions at 25 °C and used in the experiments, and normal ‘Huangguan’ pear fruits were used as a control. The rate of water loss (RWL) was calculated using the formula RWL (%) = (FW_t1_ − FW_t2_)/FWt1 × 100% (FW_t1_ = weight of the fruit at a certain storage time t1, and FW_t2_ = weight of the fruits at a certain storage time t2) [34]. Each group had 10 fruits, and three independent biological replicates were performed.

### Evaluation of brown spot disorder

According to the coverage rate of spots on the surface of pears, the BS incidence was divided into 4 levels [31]: 0 for no browning, 1 for 1 ~ 10%, 2 for 11% ~ 20%, 3 for 21% ~ 30, and 31% ~ 100%. The index of BS was calculated based on the following formula: index = Σ (number of fruit × incidence level)/ [total fruit number × 4 (the severest level)] [3].

### Metabolite statistical analysis

An advanced Xevo G2-XS QTOF mass spectrometer (Waters, UK) was used for data acquisition, and the commercial software Progenesis QI (version 2.2) (Waters, UK) and the BGI metabolomics software package metaX [65] were used for mass spectrometry data analysis (filtering out ions with a relative standard deviation (RSD) greater than 30%). Identification was based on the Kyoto Encyclopedia of Genes and Genomes (KEGG, http://www.genome.jp/kegg/) database. Significantly enriched pathways were assessed on the basis of the false discovery rate-adjusted hypergeometric test statistic (*p* ≤ 0.05). We used the prcomp function in the R software package to perform a PCA. The project uses variable importance in projection (VIP) values of the first two principal components in the multivariate PLS-DA model, combined with fold change (FC) and q-values from a univariate analysis to choose differentially expressed metabolites (DEMs) (VIP > 1 and FC > 1.2 or < 0.833 and with an adjusted q-value < 0.05 were considered significant). The cluster analysis used the pheatmap function in the pheatmap package in R.

### Transcriptome analysis of the pear exocarp

Total RNA was purified from plant tissues by ethanol precipitation and CTAB-PBIOZOL reagent according to the instructions. DNA nanoballs were loaded into the patterned nanoarray, and single-end 50-base reads were generated on the BGISeq500 platform (BGI-Shenzhen, China). Reads with low quality, connector contamination and a proportion of *N* > 5% were removed before the data analysis to ensure the reliability of the results. The selected clean reads were mapped to the reference genome of Chinese white pear (*Pyrus bretschneideri*) [1] via HISAT. Transcripts were assembled and annotated from the read alignment results by Cufflinks v2.1.1 [66]. The gene expression level was calculated based on the fragments per kilobase of transcript per million mapped reads (FPKM), and these results were further used to analyse the differentially expressed genes (DEGs) [67]. The DEGSeq method was based on a Poisson distribution, and DEG detection was carried out according to the method described in Wang L. et al. [68]. Transcripts with FC values > − 2 (upregulated) or < − 2 (downregulated) and with an adjusted *P*-value < 0.001 were considered significant. The BGI interactive reporting system (https://report.bgi.com) was used for subsequent analysis.

### Gene expression analysis by qRT-PCR

Quantitative real-time PCR (qRT-PCR) was applied to evaluate the transcription levels of genes associated with BS under different treatments. Total RNAs were extracted from collected plant materials using the TRIzol kit (Tiangen) according to the manufacturer’s instructions. qRT-PCR was conducted with the SYBR Green (Toyobo, Shanghai) in an optical 48-well plate using an ABI PRISM 7300 Sequence Detection System (Applied Biosystems, Foster City, California). Three biological replicates were performed to ensure the reliability of the data.

## Supplementary Information

**Additional file 1: Figure S1.** Show the pericarp surface differences between CK and BS. **Figure S2.** Show the PCA score plot derived from metabolite ions. **Figure S3.** Show the GO enrichment analysis of DEGs between CK and BS. **Figure S4.** KEGG enrichment analysis of DEGs between CK and BS. **Figure S5.** Show the phenotypes of BS in unbagged ‘Huangguan’ pear.

**Additional file 2.** List the number of reads based on RNA-Seq data.

**Additional file 3.** List the number of Differential metabolites between CK and BS.

**Additional file 4.** List the detailed information of genes involved in BS formation.

## Data Availability

The transcriptome datasets supporting the conclusions of this article are available in the National Center for Biotechnology Information (https://www.ncbi.nlm.nih.gov/sra/PRJNA682706). Metabolome datasets supporting the conclusions of this article are available in China National GeneBank DataBase (https://db.cngb.org /CNP0001613).

## Abbreviations

BS: Browning spot
MeJA: Methyl jasmonate
1-MCP: 1-methylcyclopropene
P: NaH_2_PO_4_·2H_2_O
ABA: Abscisic acid
GA_3_: Gibberellin A3
CTK: Cytokinin
SEM: Scanning electron microscopy
IAA: Auxin
GA: Gibberellin
JA: Jasmonic acid
SA: Salicylic acid
DAFB: Days after full bloom
DEG: Differentially expressed gene
DEM: Differentially expressed metabolite
FPKM: Fragments per kilobase of transcript per million mapped reads
FC: Fold change
RWL: The rate of water loss
PCA: Principal component analysis
PLS-DA: Plots from partial least squares discriminant
ESI+: Electrospray ionization positive ion mode
ESI−: Electrospray ionization negative ion mode
TFs: Transcription factors
VIP: Variable importance in projection
PPI: Plant-pathogen interactions
MAPK: MAPK signaling pathway
AQP: Aquaporin
qRT-PCR: Quantitative real-time PCR

## References

[1] Jun W, Wang ZW, Zebin S, Shu Z, Ray M, Shilin Z, Awais KM, Shutian T, SKS, Hao W (2013). The genome of the pear (*Pyrus bretschneideri* Rehd.). Genome Res.

[2] Yuanwen T (2011). The pear industry and research in China. Acta Hortic.

[3] Dong Y, Guan JF, Ma SJ, Liu LL, Feng YX, Cheng YD. Calcium content and its correlated distribution with skin browning spot in bagged Huangguan pear. Protoplasma. 2015;252(1):165–71. 10.1007/s00709-014-0665-5. 10.1007/s00709-014-0665-5 24965371

[4] Ma Y, Yang M, Wang J, Jiang CZ, Wang Q (2016). Application of exogenous ethylene inhibits postharvest peel browning of 'Huangguan' pear. Front Plant Sci.

[5] Guan JF (2008). Effects of fruit-bag kinds on browning spot and nutrition of ca, mg and K in Huangguan pear. J Auhui Agr Sci.

[6] Liu P, Xue C, Wu TT, Heng W, Jia B, Ye ZF, Liu L, Zhu L (2013). Molecular analysis of the processes of surface brown spot (SBS) formation in pear fruit (*Pyrus bretschneideri* Rehd. cv. Dangshansuli) by de novo transcriptome assembly. PLoS ONE.

[7] Guan JF. Influencing factors and occurrence mechanism of fruit brown spot on bagged 'Huangguan' pear fruit. Deciduous Fruits. 2008;5:15–18.

[8] Zhao SB, Wang YH, Han ZT, Geng LF, Du YM (2005). Analysis of the pathogenesis and preventive measures of ‘Jizhua’ disease of Huangguan pear. Hebei Fruits.

[9] Guan JF, Ji H, Feng YX, Li LM, Sun YL, Si JL (2006). The correlation of peel browning spot with nutrition of ca, mg, K in 'Huangguan' pears. Acta Agr Boreali Sin.

[10] Wang YT, Li X, Li Y, Li LL, Zhang SL (2011). Effect of bagging on distribution of polysaccharide and lipid in 'Huangguan' pear fruit. J Fruit Sci.

[11] Wang YT, Li X, Li Y, Li LL, Zhang SL (2011). Effects of bagging on browning spot incidence and content of different forms of calcium in ‘Huangguan’ pear fruits. Acta Hortic Sin.

[12] Wang JJ, Wang QG (2011). Effect of cold conditioning on postharvest fruit quality of 'Huangguan' pear. Food Ferment Ind.

[13] Li LM, Guan JF, Ji H, Feng YX, Sun YL, Gong XM (2008). Effects of precooling on the storage quality and peel browning of 'Huangguan' pear. Acta Agr Boreali Sin.

[14] Dan L, Cheng YD, Dong Y, Shang ZL, Guan JF (2017). Effects of low temperature conditioning on fruit quality and peel browning spot in 'Huangguan' pears during cold storage. Postharvest Bio Tec.

[15] Ma W, Fan QY, Huang LJ, Rui Ping L, Zhou ZF (2007). Investigation on the ocurrence of Jizhua disease of 'Huangguan' pear. J Heb Agr Sci.

[16] Han JH, Hong KH, Jang HI, Jo IH, Lee HJ (2002). Effect of characteristics of the bags and microclimate in the bags on russet of ‘Whangkeumbae’ pear fruit. Korea J Hort Sci Technol.

[17] Guan JF, Wenhui M, Hua JI, Yunxiao F, Limei LI, Yulong S, Liqin L (2008). Effects of bagging and fruit-expander on ca, pectin content and the quality of 'Huangguan' pear fruits. Sci Tec Rev.

[18] Xu FX, Zhang KX, Liu SY (2020). Evaluation of 1-methylcyclopropene (1-MCP) and low temperature conditioning (LTC) to control brown of Huangguan pears. Sci Hortic.

[19] Guan JF, Hua JI, Yun-Xiao F, Li-Mei LI, Yu-Long S, Jian-Li S (2005). The correlation of peel browning spot with phenolics metabolism in 'Huangguan' pears. Acta Agr Boreali Sin.

[20] Galvis-Sánchez AC, Fonseca SC, Gil-Izquierdo Á, Gil MI, Malcata FX (2006). Effect of different levels of CO_2_ on the antioxidant content and the polyphenol oxidase activity of ‘Rocha’ pears during cold storage. J Sci Food Agric.

[21] Oh KS, Lee UY, Wang MH, Hwang YS, Chun JP (2013). Increased carbon dioxide may accelerate skin browning disorder in 'Chuwhangbae' pear. Hortic Abstracts.

[22] Chengl GW, Crisosto CH (1995). Browning potential, phenolic composition, and polyphenoloxidase activity of buffer extracts of peach and nectarine skin tissue. J Amer Soc Hort Sci.

[23] Gong XM, Guan JF, Feng YX, Li LM, Sun YL (2009). Effects of calcium and boron on quality and browning spot disease of Huangguan pear. Plant Nutrition and Fertilizer ence.

[24] Fernández V, Díaz A, Blanco Á, Val J (2010). Surface application of calcium-containing gels to improve quality of late maturing peach cultivars. J Sci Food Agr.

[25] Zhang SM, Wang XG, Niu GC, Cheng-Wen Z (2001). Approach to the cause of fruit lenticel pit disease of pingguo pear ── its relationship to calcium nutrient. Northern Fruits.

[26] Liu TZ, Li XY, Li Y, Liu GS, Wang JT, Wang YB, Fu YL, Han YX (2019). Effect of calcium treatment during production on quality of stored 'Huangguan' pear fruit. Agr Biotech.

[27] Li JX, Zhou Q, Zhou X, Wei B, Ji S (2020). Calcium treatment alleviates pericarp browning of 'Nanguo' pears by regulating the GABA shunt after cold storage. Front Plant Sci.

[28] Franck C, Lammertyn J, Ho QT, Verboven P, Verlinden B, Nicolaï BM (2007). Browning disorders in pear fruit. Postharvest Biol Tec.

[29] Zhang Y, Ning X, Sun L, Xu T, Pang H, Zhai Z, Zhao Y, Xu J, Zhang Y (2018). Analysis of the content of mineral elements in‘Huangguan’pears of Jizhua disease. J Fruit Sci.

[30] Wei CQ, Ma LH, Cheng YD, Guan YQ, Guan JF (2019). Exogenous ethylene alleviates chilling injury of ‘Huangguan’ pear by enhancing the proline content and antioxidant activity. Sci Hortic.

[31] Xing DD, Mu WL, Wang QG (2013). Effect of methyl jasmonate on peel browning of postharvest 'Huangguan' pear. Food Ferment Technol.

[32] Gong XM, Guan JF, Zhang JS (2010). Effects of postharvest 1-MCP and calcium treatments on the quality and skin browning spot incidence of 'Huangguan' pear fruit. Acta Hortic Sin.

[33] González I, Cao KAL, Davis MJ, Déjean S (2012). Visualising associations between paired ‘omics’ data sets. BioData Min.

[34] Wang YZ, Dai MS, Cai DY, Shi ZB (2020). Proteome and transcriptome profile analysis reveals regulatory and stress-responsive networks in the russet fruit skin of sand pear. Hortic Rec.

[35] Hou W, Singh RK, Zhao P, Martins V, Aguilar E, Canto T, Tenllado F, Franklin G, Dias ACP (2020). Overexpression of polygalacturonase-inhibiting protein (PGIP) gene from Hypericum perforatum alters expression of multiple defense-related genes and modulates recalcitrance to agrobacterium tumefaciens in tobacco. J Plant Physiol.

[36] Medina J, Catalá R, Salinas J. Developmental and stress regulation of *RCI2A* and *RCI2B*, two cold-inducible genes of *Arabidopsis* encoding highly conserved hydrophobic proteins. Plant Physiol. 2001;125(4):1655–66. 10.1104/pp.125.4.1655. 10.1104/pp.125.4.1655 PMC88823 11299347

[37] Tyerman S, Bohnert H, Maurel C, Steudle E, Smith J (1999). Plant acuaporins: their molecular biology, biophysics and significance for plant water relations. J Exp Bot.

[38] Wang YZ, Zhang SJ, Dai MS, Shi ZB. Pigmentation in sand pear (*Pyrus pyrifolia*) fruit: biochemical characterization, gene discovery and expression analysis with exocarp pigmentation mutant. Plant Mol Biol. 2014;85(1):123–34. 10.1007/s11103-014-0173-1 24445590

[39] Rohart F, Gautier B, Singh A, Cao K-AL (2017). mixOmics: an R package for ‘omics feature selection and multiple data integration. PLoS Comput Biol.

[40] Sangprayoon P, Supapvanich S, Youryon P, Wongs-Aree C, Boonyaritthongchai P (2019). Efficiency of salicylic acid or methyl jasmonate immersions on internal browning alleviation and physicochemical quality of queen pineapple cv. "Sawi" fruit during cold storage. J Food Biochem.

[41] Seo J, Yi G, Lee JG, Choi JH, Lee EJ (2020). Seed browning in pepper (*Capsicum annuum* L.) fruit during cold storage is inhibited by methyl jasmonate or induced by methyl salicylate. Postharvest Biol Tec.

[42] Welker C, Böhm G, Schurig H, Jaenicke R (1999). Cloning, overexpression, purification, and physicochemical characterization of a cold shock protein homolog from the hyperthermophilic bacterium *Thermotoga maritima*. Protein Sci.

[43] Nordin K, Vahala T, Palva ET. Differential expression of two related, low-temperature-induced genes in *Arabidopsis thaliana*. Plant Mol Biol. 1993;21(4):641–53. 10.1007/BF00014547 8448363

[44] Ller-Uri MF (1996). Novel plant Ca-binding protein expressed in response to abscisic acid and osmotic stress. J Biol Chem.

[45] SFEH, Ibrahim FM (2015). Calcium: physiological function, deficiency and absorption. Chem Tech Res.

[46] Cabañero FJ, Martínez-Ballesta MC, Teruel JA, Carvajal M (2006). New evidence about the relationship between water channel activity and calcium in salinity-stressed pepper plants. Plant cell physiol.

[47] Maurel C, Verdoucq L, Luu D-T, Santoni V (2008). Plant aquaporins: membrane channels with multiple integrated functions. Annu Rev Plant Biol.

[48] Li HM, Wan XR, He SG (2010). Advances in plant aquaporins. Prog Biochem Biophys.

[49] Hanba YT, Mineo S, Yasuyuki H, Takahiko H, Kunihiro K, Ichiro T, Maki K (2004). Overexpression of the barley aquaporin HvPIP2/1 increases internal CO_2_ conductance and CO_2_ assimilation in the leaves of transgenic rice plants. Plant Cell Physiol.

[50] Al MGKIE, Ichimura K, Shinozaki K, Tena G, Walker JC (2002). Mitogen-activated protein kinase cascades in plants: a new nomenclature. Trends Plant Sci.

[51] Jagodzik P, Zielinska MT, Ciesla A, Marczak M, Ludwikow A (2018). Mitogen-activated protein kinase cascades in plant hormone signaling. Front Plant Sci.

[52] Zhang S, Klessig DF. MAPK cascades in plant defense signaling. Trends Plant Sci. 2001;6(11):520-7. 10.1016/s1360-1385(01)02103-3 11701380

[53] Romeis T. Protein kinases in the plant defence response. Curr Opin Plant Biol. 2001;4(5):407-14. 10.1016/s1369-5266(00)00193-x 11597498

[54] Morris PC (2001). MAP kinase signal transduction pathways in plants. New Phytol.

[55] Kumar K, Raina SK, Sultan SM (2020). Arabidopsis MAPK signaling pathways and their cross talks in abiotic stress response. J Plant Biochem Biot.

[56] Sharrock KR, Labavitch JM (1994). Polygalacturonase inhibitors of Bartlett pear fruits: differential effects on Botrytis cinerea polygalacturonase isozymes, and influence on products of fungal hydrolysis of pear cell walls and on ethylene induction in cell culture. Physiol Mol Plant P.

[57] Dong JX, Chen CH, Chen ZX (2003). Expression profiles of the *Arabidopsis* WRKY gene superfamily during plant defense response. Plant Mol Biol.

[58] Tal L, Gil MXA, Guercio AM, Shabek N (2020). Structural aspects of plant hormone signal perception and regulation by ubiquitin ligases. Plant Physiol.

[59] Zhu J, Zhang KX, Wang WS, Gong W, Liu WC, Chen HG, Xu HH, Lu YT (2015). Low temperature inhibits root growth by reducing auxin accumulation via ARR1/12. Plant Cell Physiol.

[60] Paleg SLG (1984). Low temperature-induced GA_3_ sensitivity of wheat: II. Changes in lipids associated with the low temperature-induced GA_3_ sensisivity of isolated aleurone of kite. Plant Physiol.

[61] Shepherd T, Griffiths DW (2010). The effects of stress on plant cuticular waxes. New Phytol.

[62] Koch K, Hartmann KD, Schreiber L, Barthlott W, Neinhuis C (2006). Influences of air humidity during the cultivation of plants on wax chemical composition, morphology and leaf surface wettability. Environ Exp Bot.

[63] Isabel L, Burcu B, FGL (2014). The fruit cuticle as a modulator of postharvest quality. Postharvest Bio Tec.

[64] Cui FQ, Brosché M, Lehtonen Mikko T, Amiryousefi A, Xu E (2016). Dissecting abscisic acid signaling pathways involved in cuticle formation. Mol Plant.

[65] Wen B, Mei ZL, Zeng CW, Liu SQ (2017). metaX: a flexible and comprehensive software for processing metabolomics data. BMC Bioinformatics.

[66] Trapnell C, Roberts A, Goff L, Pertea G, Kim D, Kelley DR, Pimentel H, Salzberg SL, Rinn JL, Pachter L (2012). Differential gene and transcript expression analysis of RNA-seq experiments with TopHat and cufflinks. Nat Protoc.

[67] Trapnell C, Williams BA, Pertea G, Mortazavi A, Kwan G, Baren MJ, Salzberg SL, Wold BJ, Pachter L (2010). Transcript assembly and quantification by RNA-Seq reveals unannotated transcripts and isoform switching during cell differentiation. Nat Biotechnol.

[68] Wang L, Feng Z, Wang X, Wang X, Zhang X (2010). DEGseq: an R package for identifying differentially expressed genes from RNA-seq data. Bioinformatics.

