# Supplementary material for: Transcriptome and metabolomic analysis to reveal the browning spot formation of ‘Huangguan’ pear
Source: BMC Plant Biol. 2021 Jul 3;21:321. doi: 10.1186/s12870-021-03049-8 (PMC8255024; doi:10.1186/s12870-021-03049-8)
Supplement: Supplementary file 1 — Additional file 1: Figure S1. Show the pericarp surface differences between CK and BS. Figure S2. Show the PCA score plot derived from metabolite ions. Figure S3. Show the GO enrichment analysis of DEGs between CK and BS. Figure S4. KEGG enrichment analysis of DEGs between CK and BS. Figure S5. Show the phenotypes of BS in unbagged ‘Huangguan’ pear. [file 12870_2021_3049_MOESM1_ESM.doc]

**Additional file 1**

Transcriptome and metabolomic analysis to reveal the browning spot formation of ‘Huangguan’ pear

Qi Wang^¶^, Xinyi Wu^¶^, Li Liu^¶^, Daozhi Yao, Jinchao Li, Jie Fang, Xiaonan Chen, Liwu Zhu, Pu Liu, Zhenfeng Ye, Bing Jia^*^, Wei Heng^*^


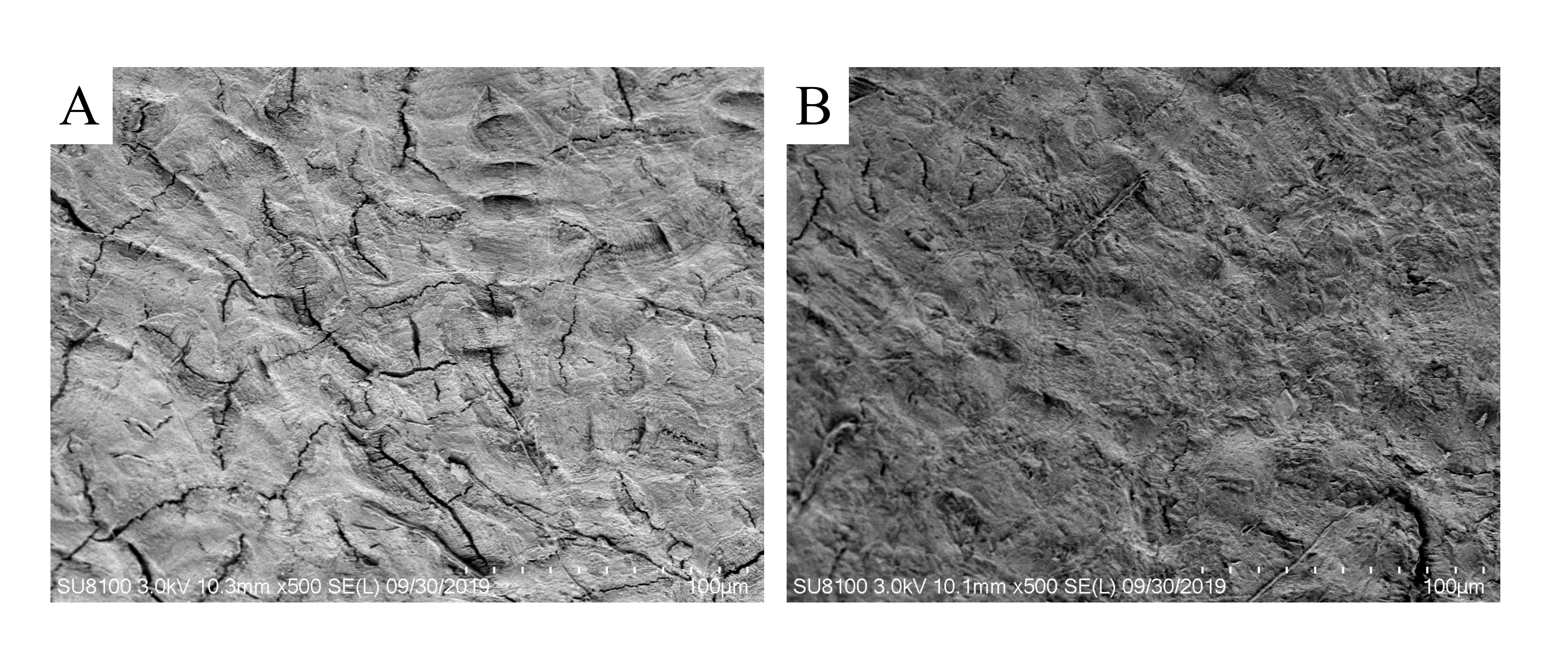


**Figure S1.**  SEM analysis of the normal part (A) and BS disease part (B) of ‘Huangguan’ pear.


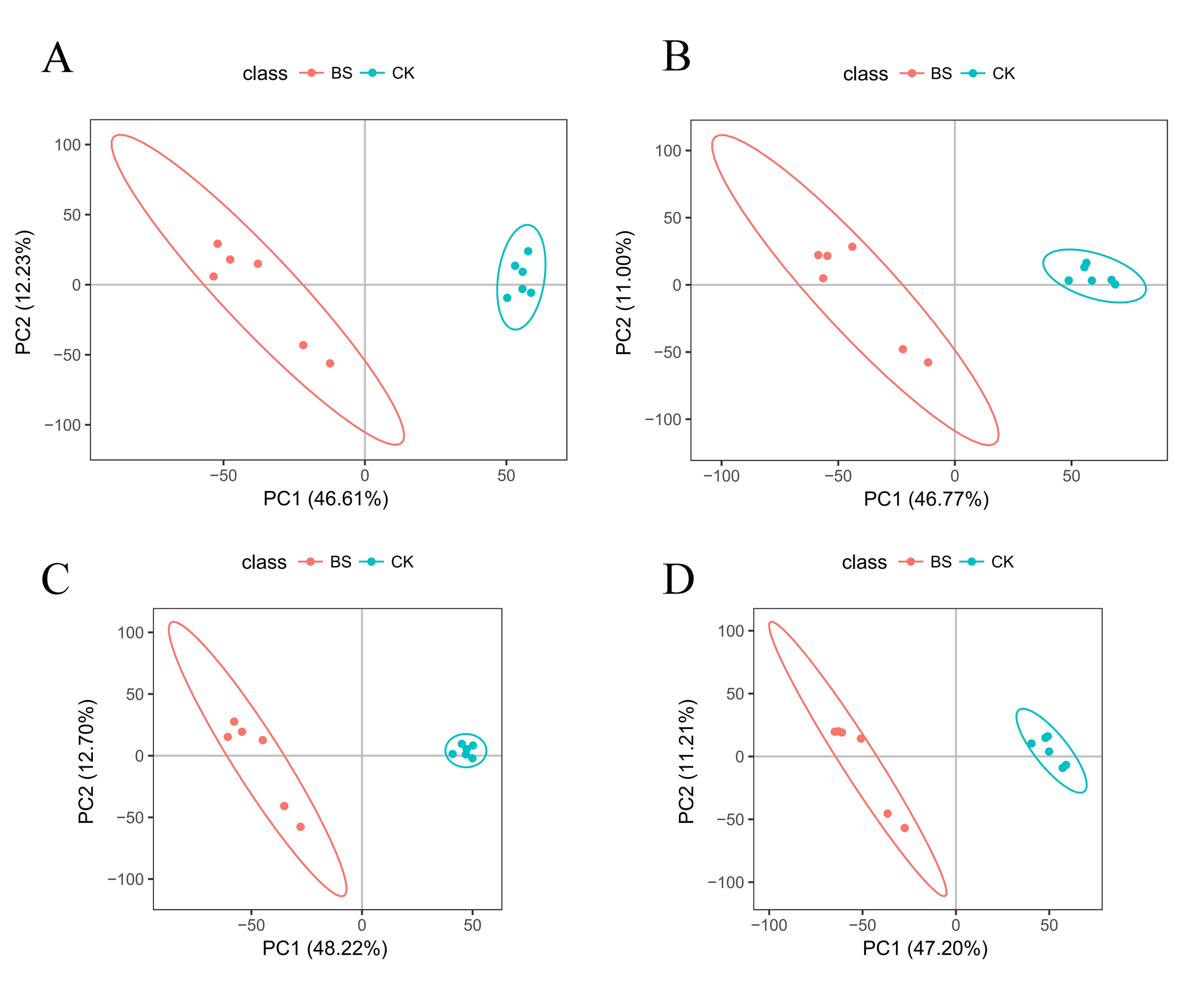


**Figure S2 Comparison of metabolites from CK and BS.** PCA score plot derived from metabolite ions acquired using ESI+ (A) and ESI− (B). PLS-DA for modeling the differences between CK and BS using ESI+ (C) and ESI− (D).


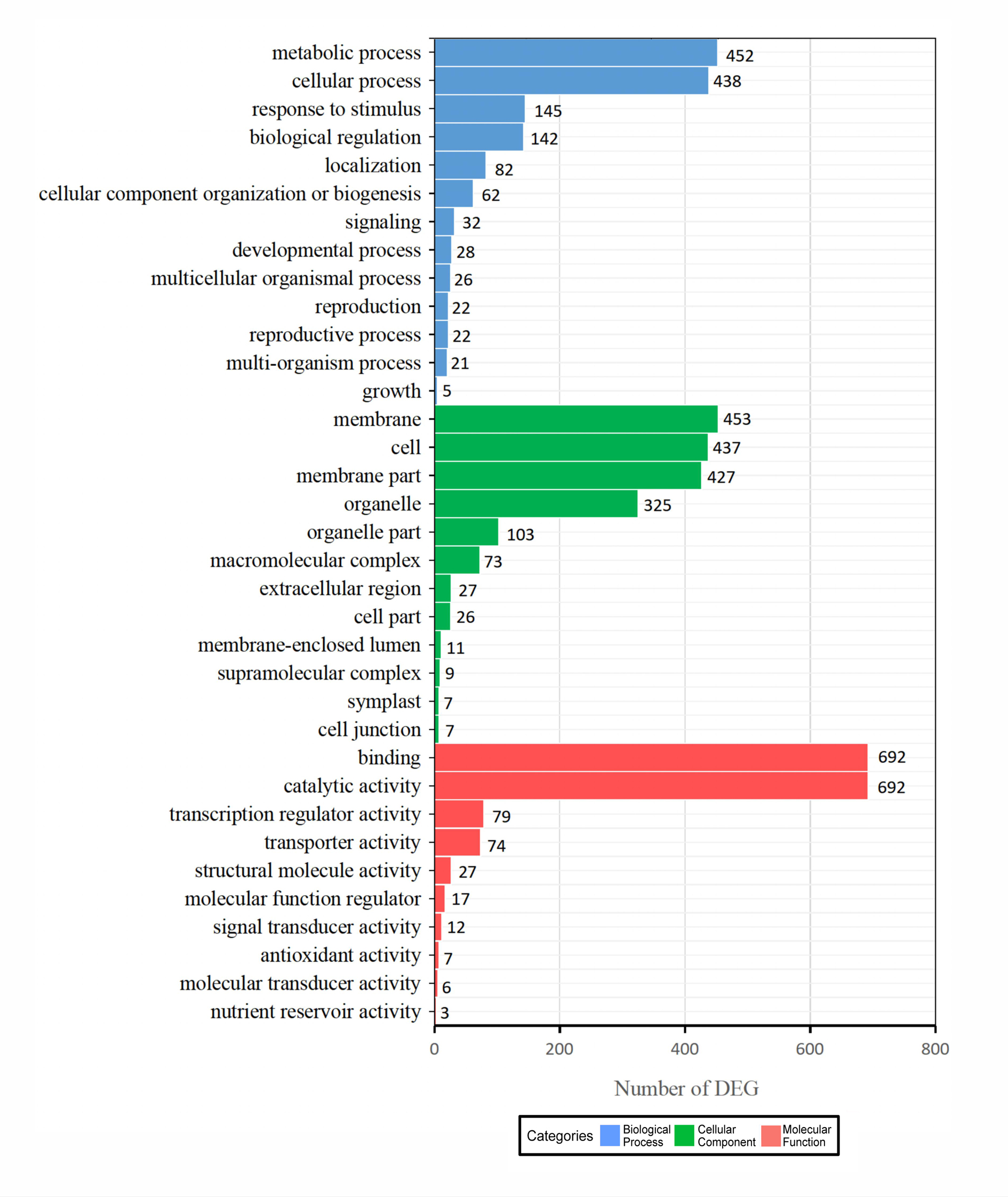


**Figure S3**. **GO enrichment analysis of DEGs between CK and BS of ‘Haungguan’ pear.** Unigenes were analyzed for GO terms that were grouped into three levels: biological process, cellular component and molecular function. The right y-axis shows the number of genes in a category.


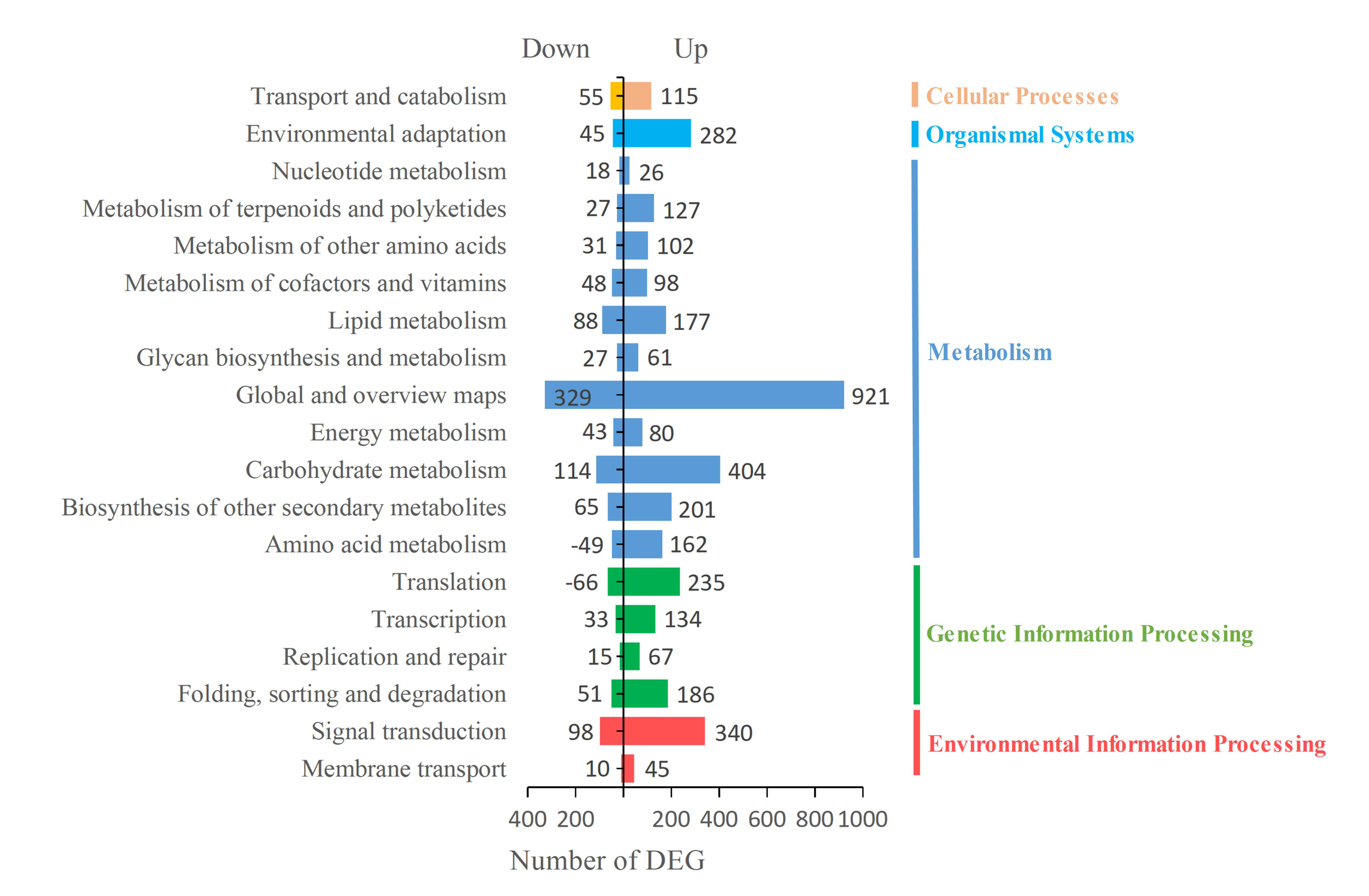


**Figure S4 KEGG enrichment analysis of DEGs between CK and BS of ‘Haungguan’ pear.** The Y-axis depicts the different pathways.


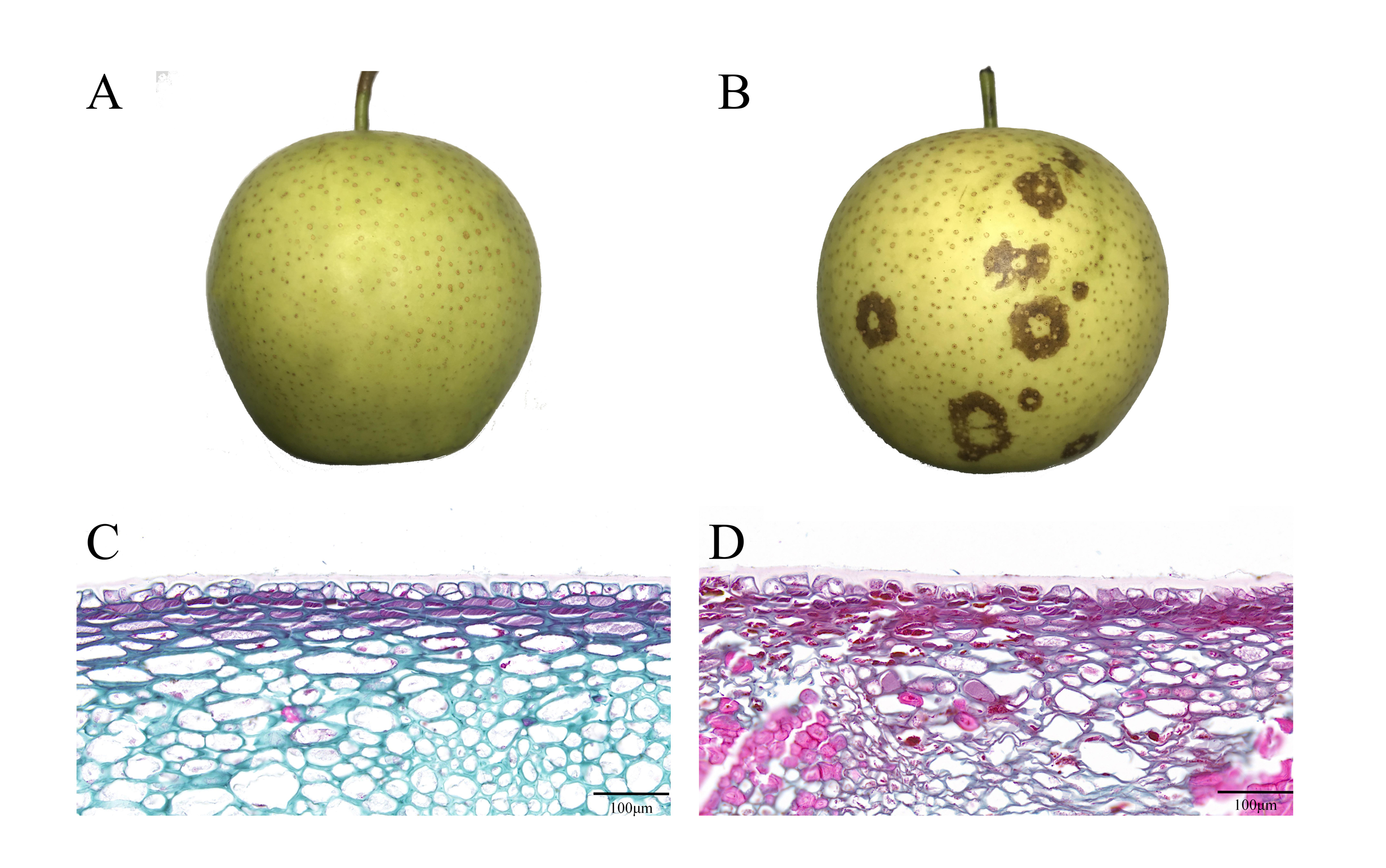


**Figure S5 Phenotypes of unbagged ‘Huangguan’ pear (A) and unbagged ‘Huangguan’ pear with BS disease (B)**. Observation of paraffin sections of the normal part (C) and BS disease part (D) of unbagged ‘Huangguan’ pear.
